# Transcriptome evolution from breast epithelial cells to basal-like tumors

**DOI:** 10.18632/oncotarget.23065

**Published:** 2017-12-08

**Authors:** Gabriel Santpere, Ana Alcaráz-Sanabria, Verónica Corrales-Sánchez, Atanasio Pandiella, Balázs Győrffy, Alberto Ocaña

**Affiliations:** ^1^ Department of Neuroscience, Yale School of Medicine, New Haven, CT 06510, USA; ^2^ Translational Research Unit and CIBERONC, Albacete University Hospital, Albacete, Spain; ^3^ Cancer Research Center and CIBERONC, CSIC-University of Salamanca, Salamanca, Spain; ^4^ MTA TTK Lendület Cancer Biomarker Research Group, Budapest, Hungary; ^5^ Semmelweis University 2nd Department of Pediatrics, Budapest, Hungary; ^6^ Regional Biomedical Research Center (CRIB), Castilla La Mancha University, Albacete, Spain; ***Correspondence to: ****Alberto Ocaña,* albertoo@sescam.jccm.es

**Keywords:** breast cancer, transcriptomic evolution, carcinoma in situ

## Abstract

In breast cancer, it is unclear the functional modifications at a transcriptomic level that are associated with the evolution from epithelial cells and ductal carcinoma *in situ* (DCIS) to basal-like tumors. By applying weighted gene co-expression network analysis (WGCNA), we identified 17 gene co-expression modules in normal, DCIS and basal-like tumor samples. We then correlated the expression pattern of these gene modules with disease progression from normal to basal-like tumours and found eight modules exhibiting a high and statistically significant correlation. M4 included genes mainly related to cell cycle/division and DNA replication like CCNA2 or CDK1. The M7 module included genes linked with the immune response showing top hub genes such as CD86 or PTPRC. M10 was found specifically correlated to DCIS, but not to basal-like tumor samples, and showed enrichment in ubiquitination or ubiquitin-like processes. We observed that genes in some of these modules were associated with clinical outcome and/or represented druggable opportunities, including AURKA, AURKB, PLK1, MCM2, CDK1, YWHAE, HSP90AB1, LCK, or those targeting ubiquitination. In conclusion, we describe relevant gene modules related to biological functions that can influence survival and be targeted pharmacologically.

## INTRODUCTION

Cancer is an evolutionary disease where the accumulation of genetic alterations leads epithelial cells to transform to premalignant lesions that ultimately may evolve to tumor cells [1, 2].

Accumulation of molecular alterations over time produces a gain of different biological functions that permits cells to proliferate, avoid programmed death, migrate or seed in distant tissue [3]. Ultimately, cells that seed and proliferate in distant organs form metastases that compromise patient life. Among these functions, deregulation of cell division and genomic instability are key characteristics of transformed cells and indeed several therapies aiming to inhibit these functions have reached the clinical setting [4]. Examples are chemotherapies that target the mitotic process or PARP inhibitors that act on the DNA repair machinery [4, 5]. Similarly, targeting of intermediate signaling nodes that are constitutively active or components involved in the regulation of protein degradation, have gained clinical approval [4, 5]. Cell metabolism including lipid metabolism are also biological functions that are necessary for the survival of tumoral cells [5]. In addition to the described alterations, the host immune response to cancer and the tumor microenvironment play a role in cancer initiation and progression [6, 7]. Finally evolution of cancer is also controlled by the interaction of transformed cells with the surrounding stroma [6, 7]. Indeed, agents targeting the tumor microenvironment and those that boost the immune response have reached the clinical practice [3, 8, 9]. The acquisition of the mentioned deregulated functions is produced at different time points in the evolution of cancer, so the identification of druggable options against these alterations could undoubtedly open avenues for the design of novel therapies.

Ductal carcinoma *in situ* (DCIS) is a lesion that can become malignant over time. It is considered as an intermediate step between breast cancer and non-transformed breast epithelial cells [10]. Indeed, in many diagnosis of breast cancer, presence of DCIS and invasive cancer coexist in the same specimen suggesting that DCIS is a preinvasive situation [10]. Therefore, the general treatment approach for this entity is surgical resection. However, it is not clear that all DCIS will progress to invasive tumors and it has been reported that some DCIS could spontaneously disappear [10, 11]. The heterogeneity of this entity highlights the importance for the identification of biological functions that could be used as predictors of progression, as this could help to optimize therapeutic options for these patients [10, 12].

DCIS is characterized by the presence of estrogen receptors or HER2 overexpression. However not all breast invasive carcinomas express both receptors. Indeed 15% of breast cancers do not express these receptors and are called triple negative cancer breast tumors (TNBC) [4, 13]. In this context, the identification of functions that are shared by DCIS and TNBC or its genomic counterpart, the basal-like subtype, could bring light into the common process of malignant transformation. In addition, it could also help to identify specific deregulated functions restricted to DCIS.

Weighted gene co-expression network analysis (WGCNA) represents a systems biology approach for studying changes across transcriptomes. It has been used to bring light into the pathogenesis of several human diseases by identifying gene modules correlated with clinical features [14] including cancer [15, 16].

In our article, we applied WGCNA to public datasets in order to reveal gene modules associated to DCIS and basal like tumors. Our study identifies several altered functions and key genes that are present in the evolution of basal like tumors from DCIS and non-transformed cells, opening the possibility to exploit them therapeutically or as biomarkers for outcome analysis.

## RESULTS

### Datasets, batch effect and principal component analysis

**Figure 1:**
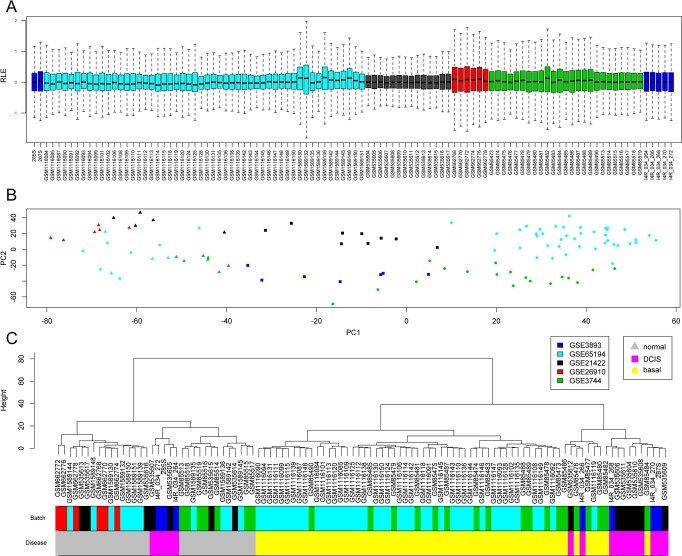
Global analysis of transcriptomes among samples In the whole panel, colors indicate the different public GEO datasets used and shape indicates clinical diagnostic. (**A**) Boxplot for all probes normalized relative log expression (RLE) values indicating no major difference between datasets. (**B**) PCA for top variable genes showing the first 2 principal components for all samples in the combined dataset, showing PC1 majorly representing differences in diagnostic. (**C**) Clustering analysis of previous PC1 values confirming that clusters mainly reflect diagnostic over differences batches.

We compiled transcriptomic microarray data from five public datasets from epithelial breast, DCIS and basal-like tumors. In order to avoid dealing with large batch confounding effects, we selected only datasets performed on the same microarray platform. We normalized all chips together and performed relative log expression (RLE) plots to determine noticeable remaining batch differences (Figure 1A). After normalization of chips from all datasets, we observed expression values with similar median and deviation, with no systematic and observable batch differences. We next assessed the transcriptomic relationships among samples by means of a principal component analysis (Figure 1B). We observed a clear separation between normal tissue samples, DCIS and basal like tumors along the PC1 axis, regardless of the dataset of origin. DCIS samples were placed between normal and basal-like samples. PC2 captured a proportion of variance explained by unknown factors that could include differences in batches. To confirm these observations, we applied a multiple lineal model to analyze the relationship between each of the first 10 principal components with the variables ‘disease’ and ‘dataset’ (Supplementary Figure 1). For PC1, the partial coefficients were statistically significant only for diagnostic categories and not for the different datasets. Dataset categories were significantly correlated to PC2 and others. Clustering analysis on samples based on PC1 values (Figure 1C) showed clustering by disease, mainly normal tissue and basal-like tumors, regardless of the dataset, except for those two datasets containing samples for one disease diagnostic only. Overall these analyses indicate that the major factor structuring transcriptomic variance among these samples correspond to disease. The combined datasets were thus considered suitable for the following co-expression network analysis.

### Gene co-expression modules correlated with breast epithelial tissue, DCIS and basal-like tumors

**Figure 2:**
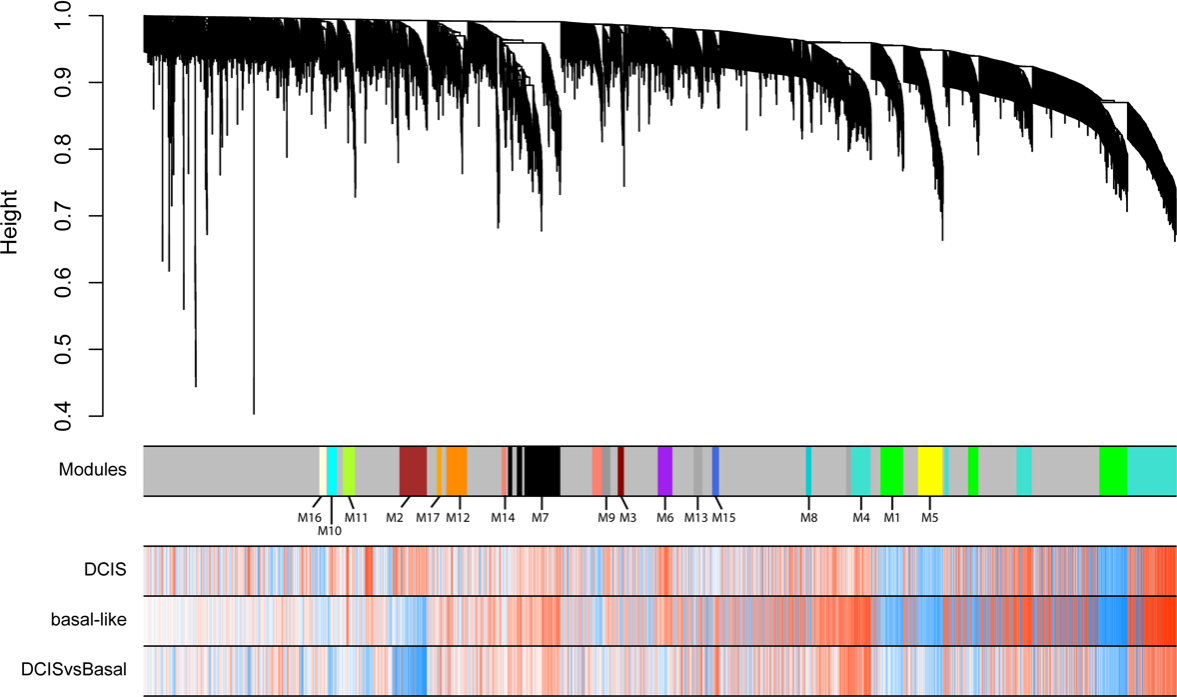
Weighted gene co-expression network analysis of the entire dataset transcriptome using top variable genes identifies 17 modules Unassigned genes were labelled in grey. Dendrogram obtained by hierarchical clustering of genes based on their topological overlap is shown at the top. Rows indicate gene correlation values with normal vs DCIS, normal vs basal and DCIS vs basal (blue indicating negative, and red positive, correlations).

**Figure 3:**
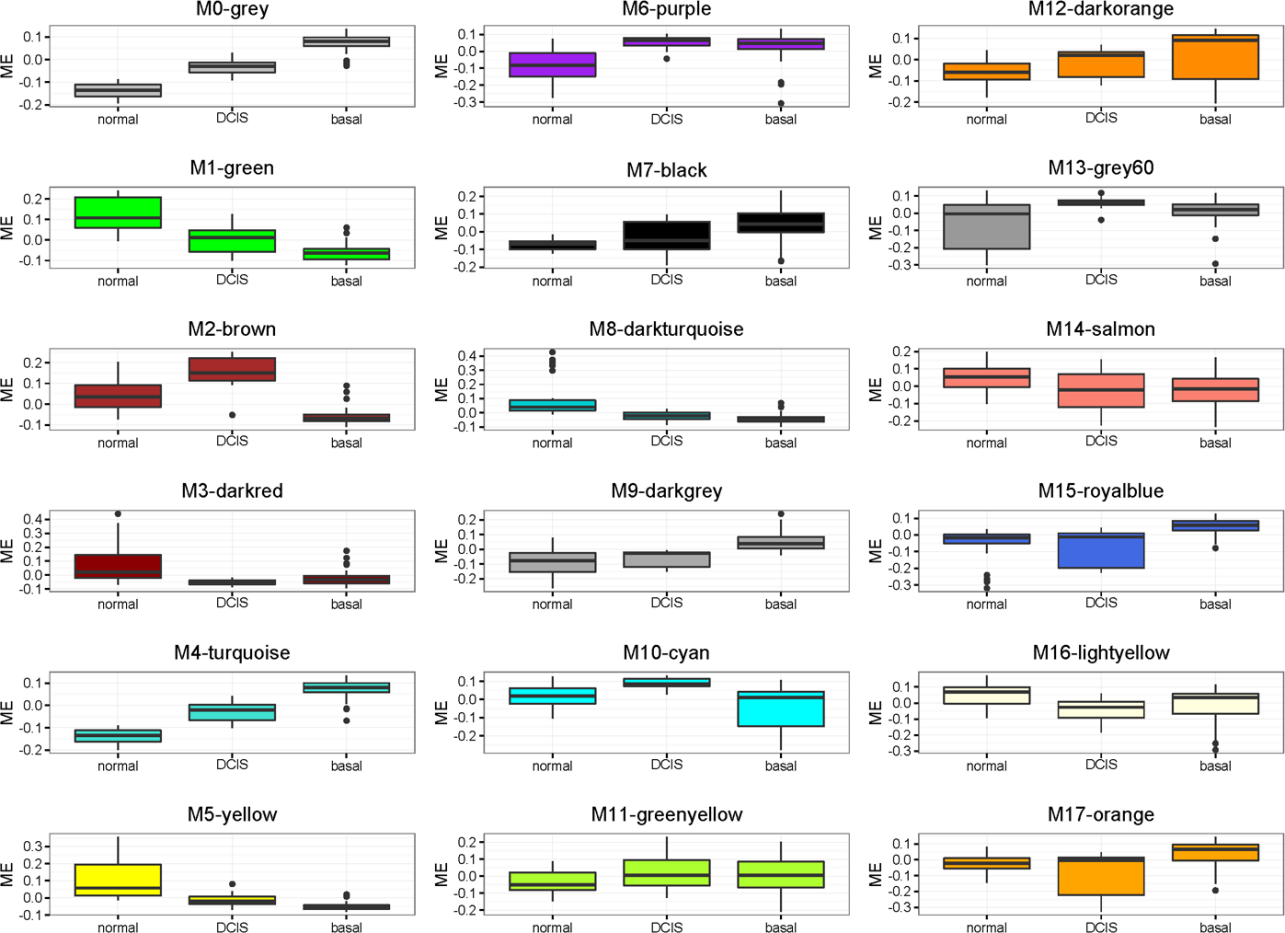
Boxplot representation of module eigengene values for the different groups

Using top variable genes in the combined dataset, we constructed a gene co-expression network by means of WGNCA. We identified 17 uncorrelated modules (Figure 2). We calculated each module eigengene, which reflects the expression pattern of all genes in a given module across samples by computing the first principal component. As we showed in the previous section, the first principal component was highly related to disease stages and not to differences among batches. We then correlated each module eigengene with normal/DCIS and normal/basal-like tumors independently (Figure 3). Eight modules (M1, M2, M4, M5, M6, M8, M9 and M15) exhibited a statistically significant correlation, considering a minimal *r* > 0.6 (Supplementary Figure 2). M4, M6, M9 and M15 showed a positive correlation in which genes tended to be up-regulated, meanwhile M1, M5 and M8 showed a negative correlation with genes mainly downregulated. Modules with a less strong correlation (*r* > 0.5) included, M2, M3, M7, M10 and M16 (Figure 3 and Table 1). Other modules showed significant association with some disease stage, but yet lower correlation values (Figure 3). M0-grey appeared also associated with disease. These is not surprising since it contains more than 3000 gene not classified in modules and we have already shown how the whole transcriptome PC1 is correlated to disease.

**Table 1:** Biological features of selected gene co-expression modules. For DCIS, we only report observations that are replicated in the two DCIS datasets analyzed

Module	Stage and main direction	#Genes	Top 5 hubs	Biological Function	PPI network	PPI hubs
M1-Green	Normal > DCIS > Basal	300	*EBF1, AKAP12, GNG11, MRGPRF, OLFML1*	Angiogenesis	57	MEOX2, CAV1, TCF4
M2-Brown	Normal > Basal	136	*MLPH, ANKRD30A, FOXA1, AGR2, ERBB4*	Gland development	18	SPDEF, RAB27B
M3-Darkred	Normal > (DCIS = Basal)	31	*CCDC144CP, MEFV, PGM5P2, OPHN1, FRG1BP*	Transport	0	
M4-Turquoise	Normal < DCIS < Basal	434	*CCNA2, MAD2L1, TPX2, UBE2T, CDK1*	Cell cycle	324	MCM2, PCNA, AURKA, CDK1
M5-Yellow	Normal > DCIS > Basal	122	*GPD1, CIDEC, PCK1, TUSC5, LEP*	Lipid metabolism	13	ALDOC
M6-Purple	Normal < (DCIS = Basal)	73	*CHMP2B, SMIM15, NRBF2, TMEM251, UBE2G1*	Lipid metabolism	8	
M7-Black	Normal < Basal	225	*CD86, FYB, SAMSN1, TFEC, PTPRC*	Immune response	86	ISG15, STAT1, HLA-C
M8-Darkturquoise	Normal > (DCIS = Basal)	26	*STARD9, SCN3B, ACSM2A, MEG3, HDC*	Respiratory chain	4	
M9-Darkgrey	Normal < Basal	67	*YWHAE, KLHDC3, TUBB, SLC25A39, PPP2R1A*	Protein localization	29	ARRB2, YWHAE, HSP90AB1
M10-Cyan	Normal < DCIS > Basal	46	*DMXL1, RNF111, ATP8B1, FAM179B, CETN3*	Protein ubiquitination	0	
M15-Royalblue	Normal < Basal	34	*PFN1, RPS7, ERI3, TOMM20, SSB*	Metabolism and RNA processing	9	RPS7

By comparing DCIS against basal-like tumors we found a subset of the mentioned modules to be progressively associated with the two assessed stages, showing intermediate eigengene values in DCIS between normal and basal-like samples. This was the case for modules M1, M4 and M5. Also, interestingly, other modules suggested specific changes in DCIS samples not shared by basal-like tumors, particularly in M2 and M10. Finally, we observed modules that apparently were only dysregulated in basal-like samples, such as M15 or M9.

Genes within a module that show highest correlation with the module’s eigengenes can be considered hub genes [17]. A list of the top hub genes in each of the above-mentioned modules is presented in Table 1.

Since we had a lower DCIS sample size we decided to replicate DCIS findings using an alternative, yet also small, dataset including 6 normal and 19 DCIS samples analyzed in a different microarray platform. We calculated the eigengenes of the previous gene modules produced by grouping the corresponding genes, when present, in this new dataset. Modules eigengenes were highly and consistently correlated with disease in modules M4, M5, M10 and M12. Other previously disease-associated modules showed non-significant or different trends in this second dataset for DCIS (Supplementary Figure 2).

### Overlapping of genes with previous published studies

We set up to compare our findings with published analysis contrasting normal and DCIS and/or basal-like tumors. We compared the genes found in our modules with genes previously reported to be de-regulated in DCIS in two separate studies. In particular, we gathered a list of up-regulated and down-regulated genes from Lee, *et al.* 2012 [18] analyzing the progression from DCIS to invasive breast cancer. In our filtered datasets, we evaluated 62 (39 up- and 19 down-regulated) of the 74 genes that were reported [18]. Their reported up-regulated genes were found enriched in our M1 and M11 modules (Fisher *P*-value < 0.05) (Supplementary Table 1). The two modules are composed by genes mainly up-regulated in DCIS, basal samples or both. Notably 24 of the 39 up-regulated genes were found in the M11 module, mainly representing molecules related to cell adhesion and extracellular matrix. Six genes related to the immune response were also found in our M7 module (Supplementary Table 1). We then studied the overlap between our modules and a set of >1000 genes with high discriminant coefficients between different stages of breast cancer progression, described in Ma XJ *et al.*, 2003 [19]. We found a significant overlap (Fisher *P*-value < 0.05) in M4 (cell cycle) and M5 (lipid metabolism) modules, which were the top two modules in terms of consistency across datasets and strength of association with breast cancer (Supplementary Table 1). We also compared our data with the result of applying WGNCA on microarrays derived from different breast cancers in Clarke *et al.*, 2013 [20]. The authors of this study identified two co-expression modules associated to survival in basal-like tumor samples. We found that genes in one of their modules significantly overlapped with genes in our M7-black and M14-salmon modules (Supplementary Table 1). The overlap with M7-black was notable, with 139 of the 225 genes. The other module in Clarke *et al.*, 2013 [20] significantly overlapped with four of our modules. The top ones being M9-darkgrey and M4-turquoise, both of them strongly correlated with the basal-like stage in the present study.

Finally, we downloaded and processed expression data from the Metabric study [21–23] (see material and methods section). We compared expression levels of genes in each module between normal and tumors samples (Supplementary Table 2). With the exception of the M14 signature, each module had a significant expression change when comparing the normal and cancer samples. We have to note, that although significant, the expression fold change was less than 5% for 7 of the 17 gene sets. The highest up-regulation was present in the green M5, M9, and M2 signatures. At the same time, only the M1 and M5 signatures had a lower expression in tumor samples. This direction of change was consistent in our original dataset in which M1 and M5 showed the strongest down-regulation in pathological samples compared to normal tissue (Supplementary Table 2).

### Gene ontology enrichment analyses

To get insights into the biological functions that are implicated in the evolution of non-transformed cells into basal-like breast cancer, we performed Gene Ontology (GO) enrichment analysis focused on biological process categories for genes in each module (shortly summarized in Table 1, fully detailed in Supplementary Table 3, and illustrated in Supplementary Figure 3 using ReviGO).

M1 genes, mainly downregulated in basal-like tumors, were overrepresented in categories related to angiogenesis and cell adhesion, among others. M2, mostly downregulated in basal-like samples, contained genes involved in gland development, including among others 3 genes related to lactation (*ERBB4*, *XBP1*, *ATP7B*). M3 was a small module of mainly down-regulated genes enriched in vesicle transport categories. The M4 module mainly included genes that were progressively upregulated in DCIS and basal-like tumors and were related to cell cycle, nuclear division, DNA replication and cell division (Supplementary Figure 3). Genes within M5 module were progressively changing expression from normal to DCIS to basal-like stages and main over-represented functions included lipid storage and lipid metabolism, among others (Supplementary Figure 3). An interesting enriched function observed in this module was the execution phase of apoptosis. Lipid metabolism was also enriched in M6, but in this case genes were mostly up-regulated (Supplementary Figure 3). An up-regulation of genes involved in the immune response was observed in the M7 module (Supplementary Figure 3). Relevant genes in this function included CD86, essential for T-lymphocyte proliferation and interleukin-2 production, by binding to CTLA-4; or PTPRC a positive regulator of T-cell coactivation upon binding to DPP4 (Table 1). M8 contained and enrichment of genes related with the electron respiratory chain (Supplementary Figure 3). Functions included in the M9 module, mainly composed by upregulated genes in basal-like tumors, included establishment of protein location, small GTPase mediated signal transduction or cellular component organization, among others (Supplementary Figure 3). M10, specifically upregulated in DCIS samples showed enrichment in ubiquitination or ubiquitin-like processes and the transforming growth factor beta (TGFbeta) pathway, among others. M15 genes, mainly upregulated in basal-like tumors, were generally enriched in metabolism categories including pentose-related processes (Supplementary Figure 3). Finally, M12 was found enriched in functions related to cell adhesion, vascular development and extracellular matrix.

### Protein-protein interaction networks

**Figure 4:**
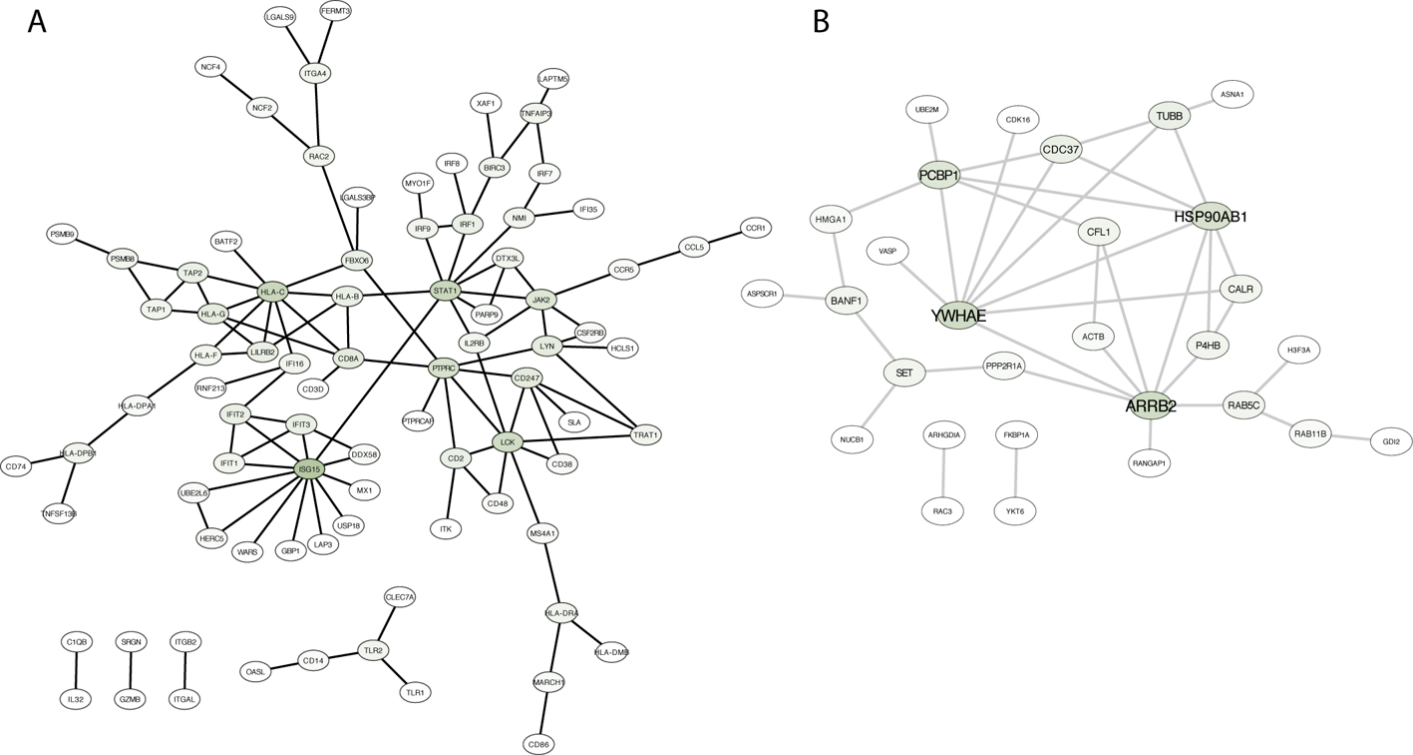
Protein-protein interaction network based on direct physical interaction among genes in two different modules (M7 in a, and M9 in b) Darker green in nodes indicates higher degree.

To further characterize functional pathways within modules, we intersected genes in modules with the physical protein-protein interactions (PPI) network from BioGrid. Subnetworks of genes were obtained (Table 1) using the function induced subgraph from the R library rTRM [24] and networks were visualized using Cytoscape [25]. We first focused on the M7 module as it is associated with immune response, and therapies to modulate the immune system are currently approved and deeply investigated in several solid tumors. Figure 4A shows the network in which HLA-C, ISG15 and STAT1 play a relevant role as the highest connected nodes. The angiogenesis-related module M1 produced a PPI subnetworks mainly nucleated around MEOX2, CAV1 and TCF4 (Supplementary Figure 4).

Next, we focused on the M9 module where protein location and cellular component organization were the principal enriched functions. PPI subnetwork identified HSP90AB1, YWHAE, PCBP1 and ARRB2 as the key interaction proteins (Figure 4B). This observation highlights the importance of protein organization in tumors to maintain the survival of cancer cells. In the M4 we observed an important number of interacting proteins linked with cell division and DNA replication such as MCM2, PCNA, AURKA and CDC2, among others (Supplementary Figure 4). No first order physical interactions were retrieved from M10 module.

### Druggable opportunities and association with clinical outcome

Next, we explored druggable proteins within the most relevant PPI networks. Druggable proteins within the M4 network include cell cycle kinases and regulators such as AURKA, AURKB, PLK1, MCM2 or CDK1, among others. For M9 module YWHAE and HSP90AB1 can be pharmacologically inhibited, and for LCK in the M7 module. Of note, compounds against pathways within a module are currently under evaluation as those targeting ubiquitination (ISG15 gene) or STAT1, as observed in the M7 module. Supplementary Table 4 describes the complete list of available drugs against the key network connecting proteins and top hub genes for each module.

**Figure 5:**
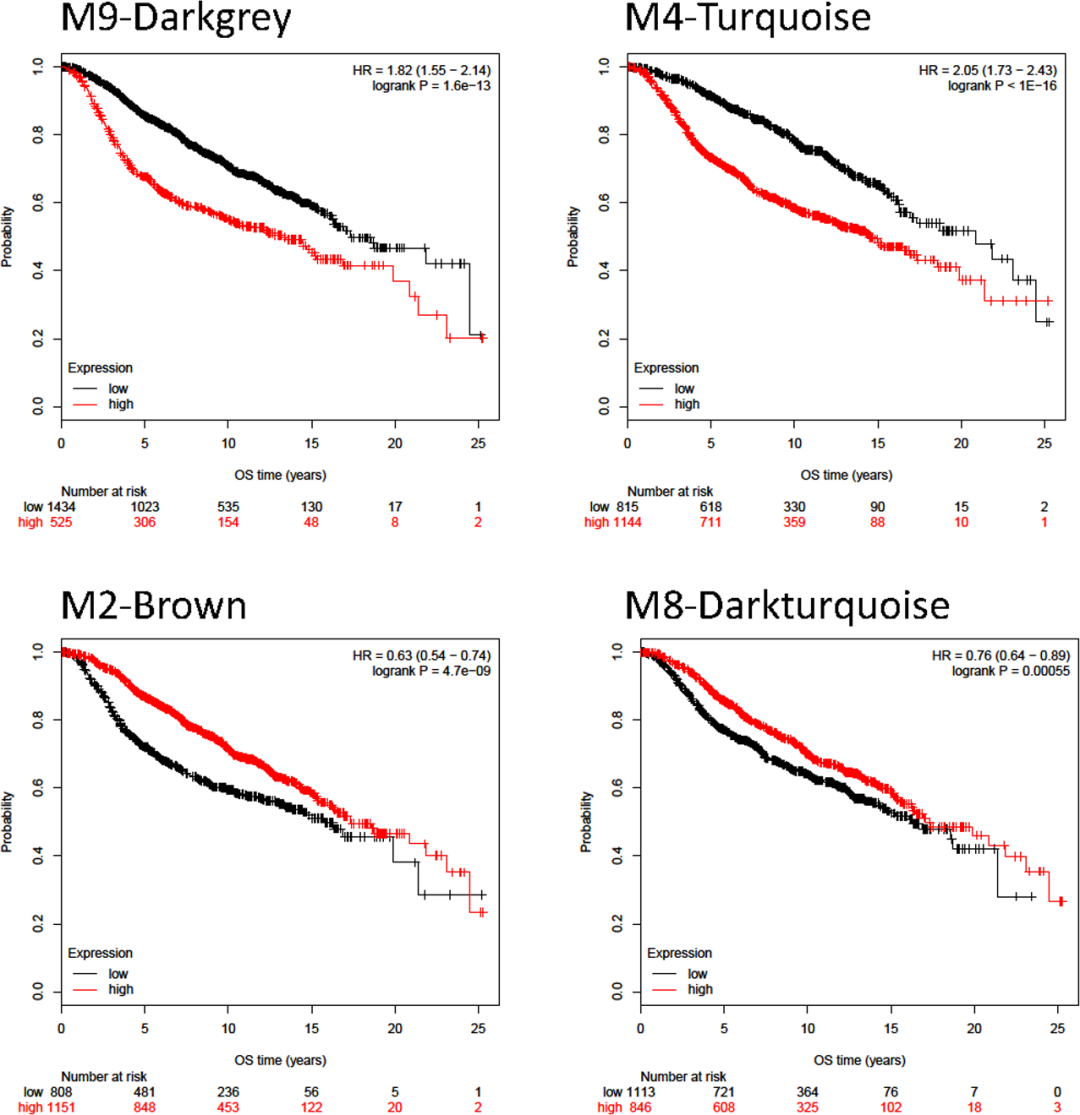
Kaplan-Meyer survival curves for modules showing the strongest differences between patients with high and low expression values in the Metabric dataset

Finally, we explored the association of the genes in each module with clinical outcome of patients in the Metabric dataset [21]. We hypothesized that those genes linked with detrimental outcome could have a relevant role in the oncogenic process and by contrary, if linked with beneficial outcome could be associated with a host defense mechanism. Overall survival was computed using samples provided by the Metabric study [21]. The best performing gene sets capable to influence survival were the M2, M9, M4, and M8 gene sets. In these, when plotting the *p*-values versus the cutoff, almost all cutoff values delivered a significant correlation (Supplementary Figure 5). Higher expression of the signature correlated to better survival in the M2 and M8 modules, while lower expression of the signature correlated to better survival in the M9 and M4 signatures. Some signatures including the M14, M17, M11, M13, M3 and M12 were not prognostic. Kaplan-Meier survival plots for a selected set of signatures is presented in Figure 5, and the achieved hazard rate and *p*-values for each dataset is displayed in Supplementary Table 5.

## DISCUSSION

In the present article, we describe modules of genes that change in DCIS and basal-like tumors with respect to normal breast. Our intention was to identify biological functions and networks of interacting proteins, relevant in the evolution to basal-like breast cancers that could potentially be inhibited pharmacologically. In addition, we observed that genes contained within some of these functions were strongly linked with clinical outcome.

M1, M2, M3 and M5 genes, where mainly downregulated in basal-like tumors over representing categories related to angiogenesis, cell adhesion, gland development, vesicle transport and lipid storage or metabolism. Basal-like tumors are characterized by its dedifferentiation and metastatic capacity, with a specific pattern of relapse [26]. Therefore, downregulation of genes related to cell adhesion or gland development seems to be in line with the current process of cancer progression. The global downregulation of the module enriched in angiogenic related genes confirm the limited role of angiogenesis in breast cancer, reinforcing the lack of efficacy observed with antiangiogenic therapies in this disease [4, 27]. An interesting function is the upregulation of genes related to the lipid metabolism, as observed in the M6 module. This finding is in line with recent studies describing the role of lipid metabolism genes in association with the initiation of metastases [28].

In the module 4 we identified genes that were progressively upregulated in DCIS and basal-like tumors and were mainly related to cell cycle/division, and DNA replication. This finding is not surprising as basal-like tumors have a high proliferation rate and present genomic instability, therefore agents targeting mitosis and producing DNA damage have clinical efficacy [4]. Of note, some of the genes codify for proteins that have a relevant presence in the PPI network analysis like PLK1, AURKA/B, CDK1, MCM2 or PCNA. Interestingly some are druggable kinases involved in the regulation of mitosis like PLK1 or AURKA/B. It should be mentioned that drugs against some of these proteins are currently in clinical development in different solid tumors, but not in basal-like tumors [29]. It could be expected that some of these kinases were associated with poor clinical outcome. Consistently with this idea, our survival curves showed that relatively low expression of genes in M4 predicts increased survival.

In module 7 we observed upregulation of genes related to the immune system. Activation of the immune system is a therapeutic strategy against cancer that has reached the clinical setting with the incorporation of check-point inhibitors to the current armamentarium [8, 9, 30]. Check point inhibitors pretend to activate the immune system provoking a response against the tumor [30]. In this context, one of the top hubs includes CD86 that is the ligand of the cytotoxic T-lymphocyte-associated protein 4 (CTLA-4) [31]. Finally, the identification of HLA components suggests that this family of proteins can have a relevant role in the activation of the immune response by presenting antigens to effector lymphocytes [31]. Globally, our findings reinforced the role of the immune system in basal-like tumors, supporting the current development of this type of agents in this indication.

In M9 module we identified upregulated genes in basal-like tumors related to protein location, small GTPase mediated signal transduction or cellular component organization. Protein location is essential when cells have a high proliferating rate like is the case of basal-like tumors [3]. In line with this, lower expression was associated with better survival. This module was found also highly correlated in DCIS in the replica dataset. The PPI network analysis reveals relevant proteins included in this function like HSP90, YWHAE, or proteins that act on ubiquitination. Deubiquitinating agents are current in preclinical stage of drug development, and HSP90 inhibitors are in different stages of clinical development [32]. Of note ubiquitination or ubiquitin-like processes where specifically upregulated in DCIS as observed in module M10.

M4 and M9 genes were expressed at lower levels by patients with higher survival rates. This makes of them a logical therapeutic target for inhibition. Indeed, we found druggable opportunities among proteins observed in the PPI network analyses for these two modules. Some targets included AURKA, AURKB, PLK1, MCM2 or CDK1, from M4, and YWHAE and HSP90AB1 in the M9 module.

In conclusion, we have identified modules of genes that have changed between normal breast tissue, DCIS and basal-like tumors. Our findings identify novel functions at a transcriptomic level which are potentially druggable, and therefore suggest therapeutic opportunities. The identification of distinct cellular functions such as regulation of protein location, activation of the immune system, cell cycle or DNA replication suggest potential therapeutic combinations, like the concomitant administration of checkpoint and HSP90 inhibitors or deubiquitinating agents; or agents acting on cell division or DNA with chaperones or immunomodulators. Evaluation of these combinations in animal models is a future step.

## MATERIALS AND METHODS

### Selection of datasets from public databases

We screened the Gene Expression Omnibus (GEO) database from NCBI for raw microarray data derived from samples of normal epithelial breast tissue, DCIS and basal-like tumors. To avoid difficulties produced by cross-platform comparisons, we mined datasets using the same chip platform. We downloaded in total 29 normal tissue, 16 DCIS and 59 basal-like tumor samples from five studies with the following GEO accession numbers: GSE21422, GSE26910, GSE3744, GSE3893 and GSE6519, all of them loaded onto an Affymetrix Human Genome U133 Plus 2.0 Array. We downloaded and additional dataset (GSE33692) of 6 normal tissue and 19 DCIS to check replicability. This second dataset was produced using the Affymetrix Human Exon 1.0 ST Array.

### Data normalization and weighted gene co-expression network analysis (WGCNA)

Microarray data analyses were performed using R Bioconductor packages. CEL files we read and normalized together using the robust multichip average (rma) algorithm [33]. Expression of all probes for each gene were collapsed by calculating the average value using the collapseRows function [34]. Standard deviation of expression among samples was calculated for each gene and we retained for further analysis only those belonging to the last quartile. Principal component analysis was performed using the R function *prcomp*. We constructed a gene co-expression network with all samples using the R implementation of the WGCNA method [35]. A gene co-expression network was constructed from a Pearson correlation matrix between all genes that was then converted into an unsigned adjacency matrix applying a power function with a customizable power parameter. We used a soft-power threshold of 6 after assessing the goodness of fit into a scale-free topology network trying a range of values. Modules were identified using hierarchical clustering of a dissimilarity measure derived from a topological overlap matrix (TOM) [17]. We calculated module eigengenes (first principal component of the modules gene expression across samples) and highly correlated genes (Pearson *R* >/=0.8) were merged obtaining a total of 17 modules labelled with colors and numbers. Genes not assigned to any module were labeled as ‘M0-grey’.

### Gene ontology enrichment analysis and protein-protein interaction network

Gene Ontology enrichment analysis was performed using GOstats [36]. Physical protein-protein interactions were obtained from BioGrid latest release. Subnetworks of genes were obtained using the function *induced_subgraph* implemented in R package rTRM [24]. Network analysis and visualization were performed using Cytoscape [25].

### Processing of the metabric dataset

All together 1,988 cancer samples measured by Illumina gene chips published in the Metabric project were obtained from the European Genome-phenome Archive (EGA) (https://www.ebi.ac.uk/ega/) [23]. As a substitute of using the processed dataset, the entire dataset including each individual arrays was re-processed. In this, the raw expression data were imported into R (https://www.r-project.org/) and summarized using the beadarray package [37]. For annotation, the illuminaHumanv3 database of Bioconductor was used (http://www.bioconductor.org). All unmapped probes were removed during summarization (*n* = 319). At the next step, a quantile normalization was completed using the preprocessCore package (https://github.com/bmbolstad/preprocessCore). Finally, a scaling normalization was performed to set the mean expression on each array to a pre-defined value. Several genes had multiple probes for a given gene–in these cases the one with the highest span of detection range was utilized.

### Association with clinical outcome

Survival was analyzed by Cox regression, and Kaplan-Meier plots were drawn to visualize the results. Cox regression analysis was performed using the “survival” R package v2.38 downloaded from CRAN (https://cran.r-project.org/web/packages/survival/index.html). Kaplan-Meier plots were generated applying the “surviplot” R package v0.0.7 (http://www.cbs.dtu.dk/∼eklund/surviplot/). Cutoff value for the survival analysis was determined by running the analysis using each percentile between the lower and upper quartiles of expression as thresholds to dichotomize the patients as described previously [38]. There were all together 144 normal samples in addition to the cancer samples. Gene expression comparing normal and tumor samples was computed by a Mann-Whitney test.

### Gene-drug interactions

For the evaluation of compounds that could potentially interact with the identified genes we used the Drug Gene Interaction Database (DGIdb) (www.dgidb.genome.wustl.edu).

## SUPPLEMENTARY MATERIALS FIGURES AND TABLES








