# Supplementary material for: Transcriptome evolution from breast epithelial cells to basal-like tumors
Source: Oncotarget. 2017 Dec 8;9(1):453–63. doi: 10.18632/oncotarget.23065 (PMC5787480; doi:10.18632/oncotarget.23065)
Supplement: Supplementary file 1 [file oncotarget-09-453-s001.pdf]

Transcriptome evolution from breast epithelial cells to basal-like tumors

SUPPLEMENTARY MATERIALS

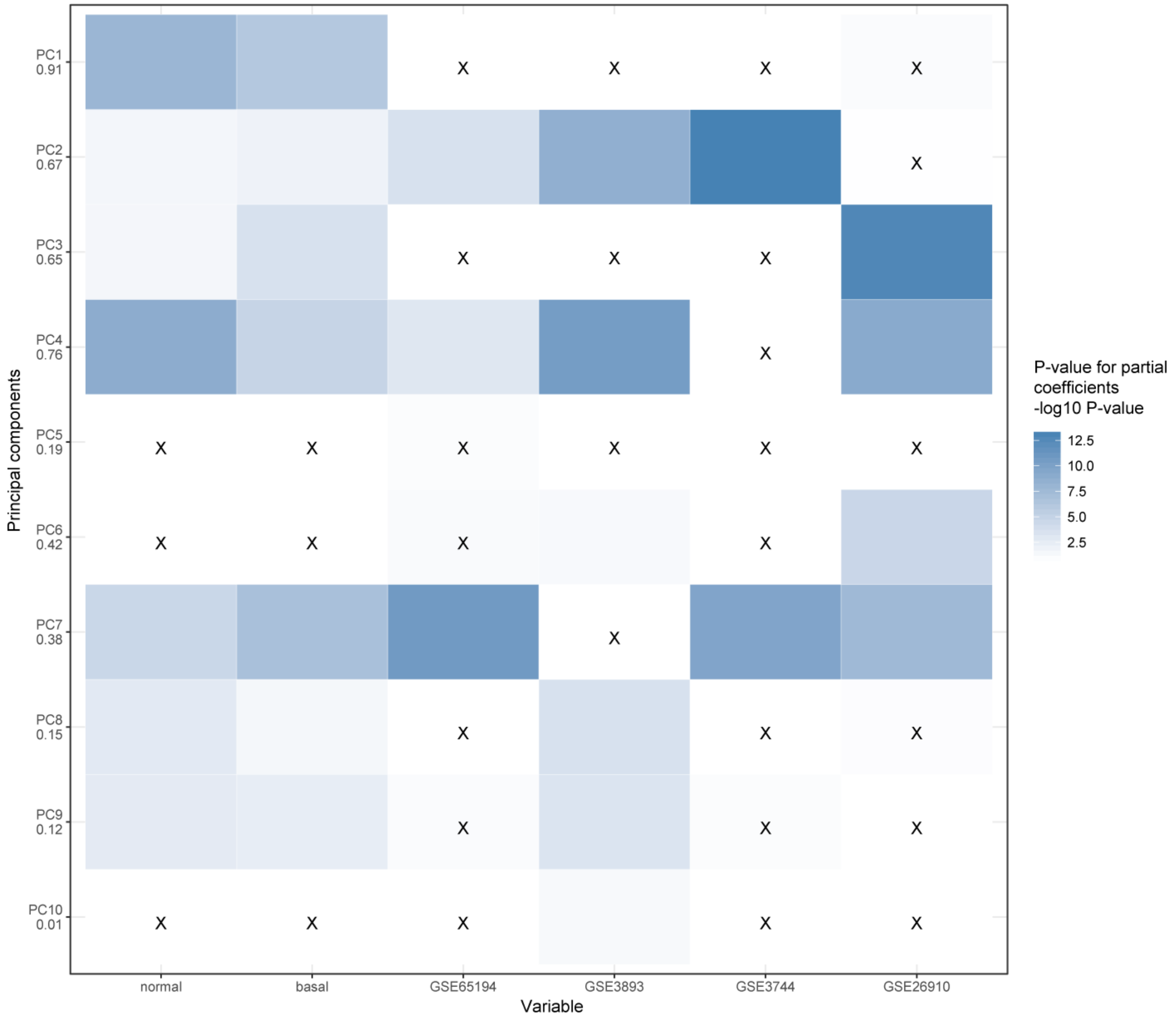

Supplementary Figure 1: Heatmap representation of the –log10 *P*-values for the partial coefficients of the multiple lineal model of each one of the 10 first principal components, predicted by Disease and Batch variables.

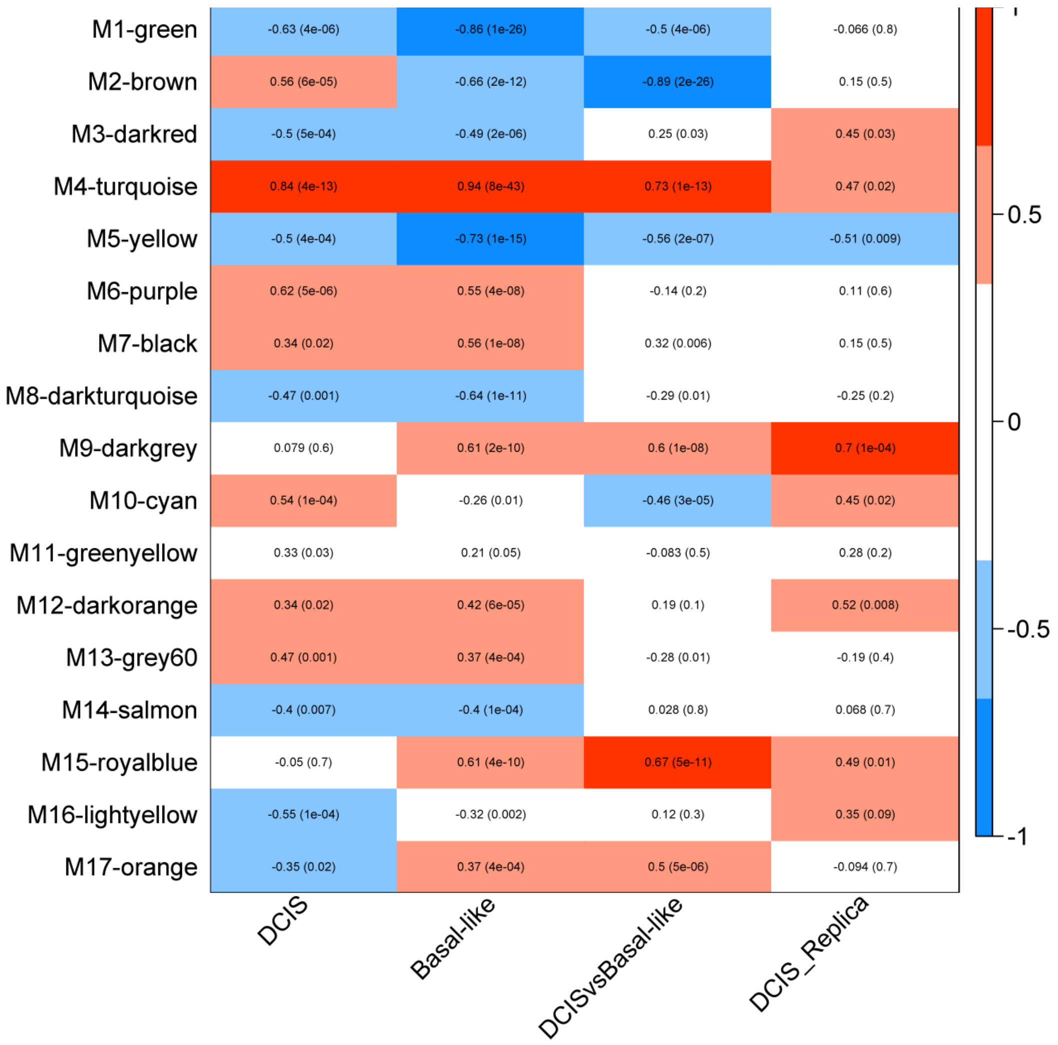

Supplementary Figure 2: Heatmap showing correlation and *P*-values for each module eigengene and disease categories. Last column uses module eigengene values from matched genes from another external dataset of DCIS and normal samples.

M7-Black

REVIGO Gene Ontology treemap

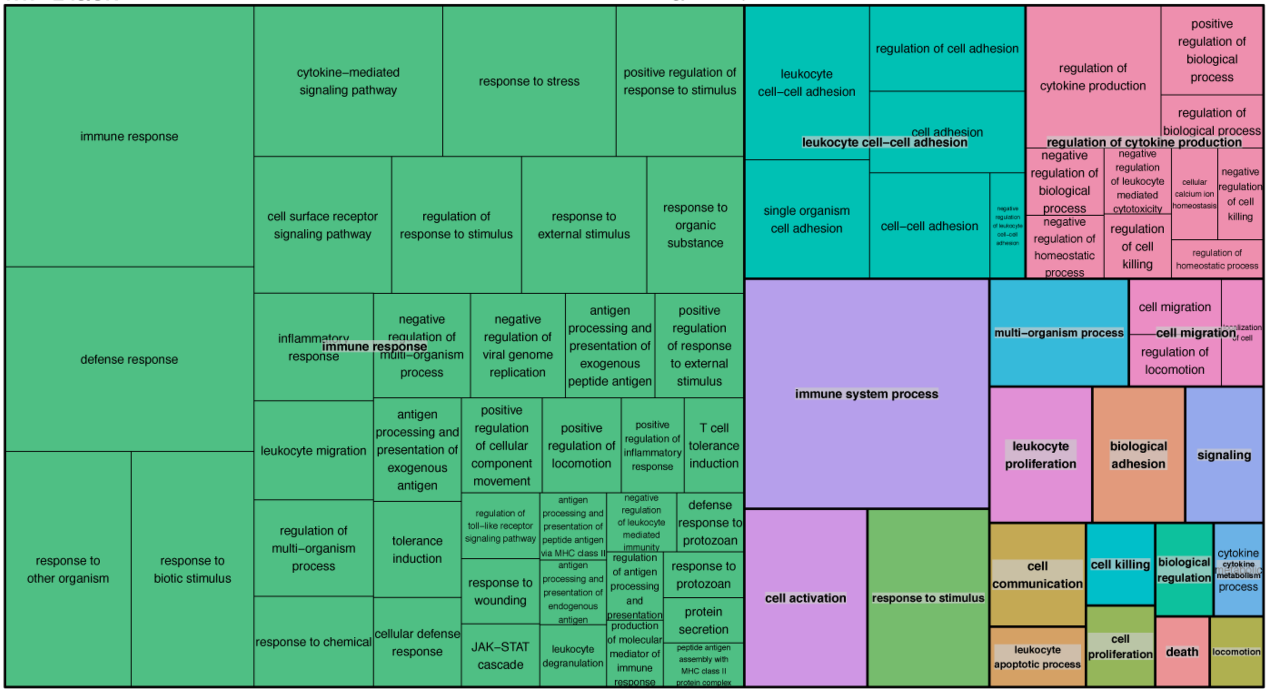

M2-Brown

REVIGO Gene Ontology treemap

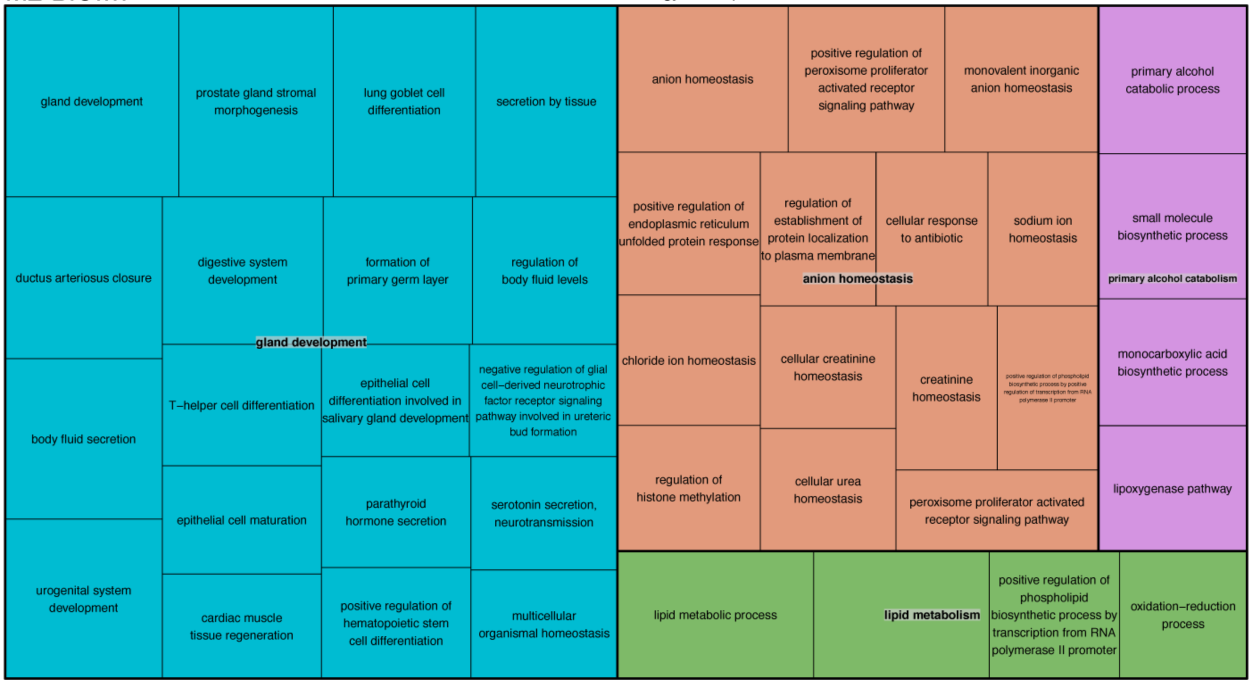

M10-Cyan

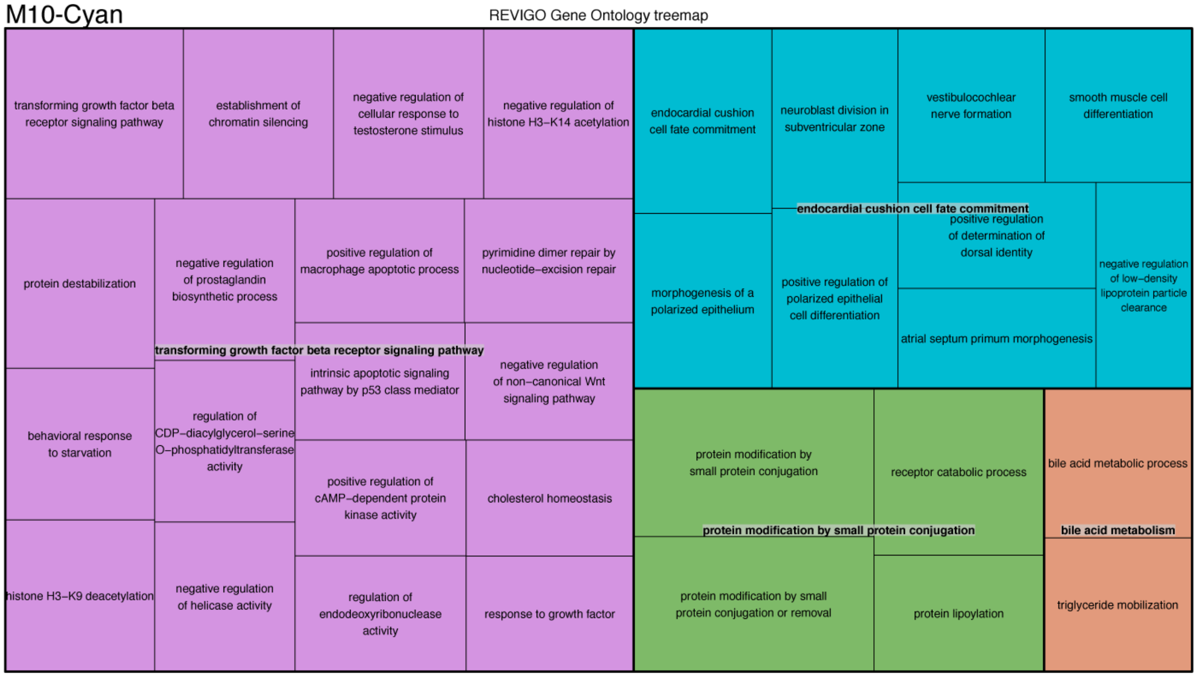

M9-Darkgrey

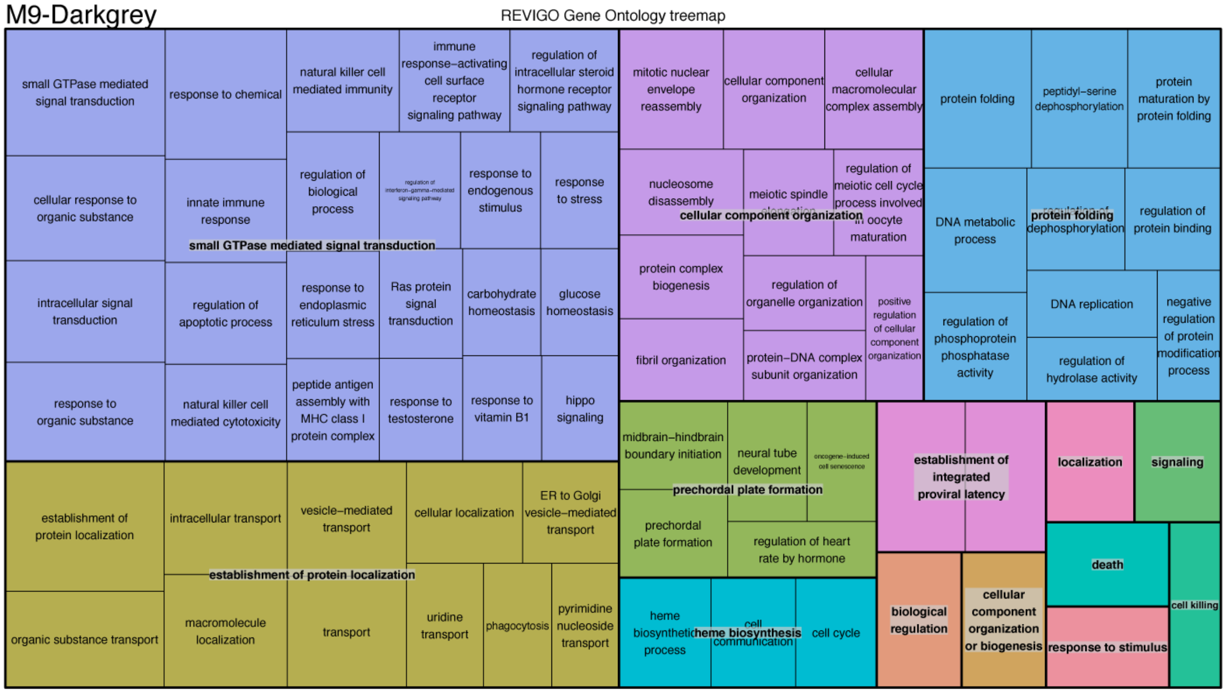

M12-Darkorange

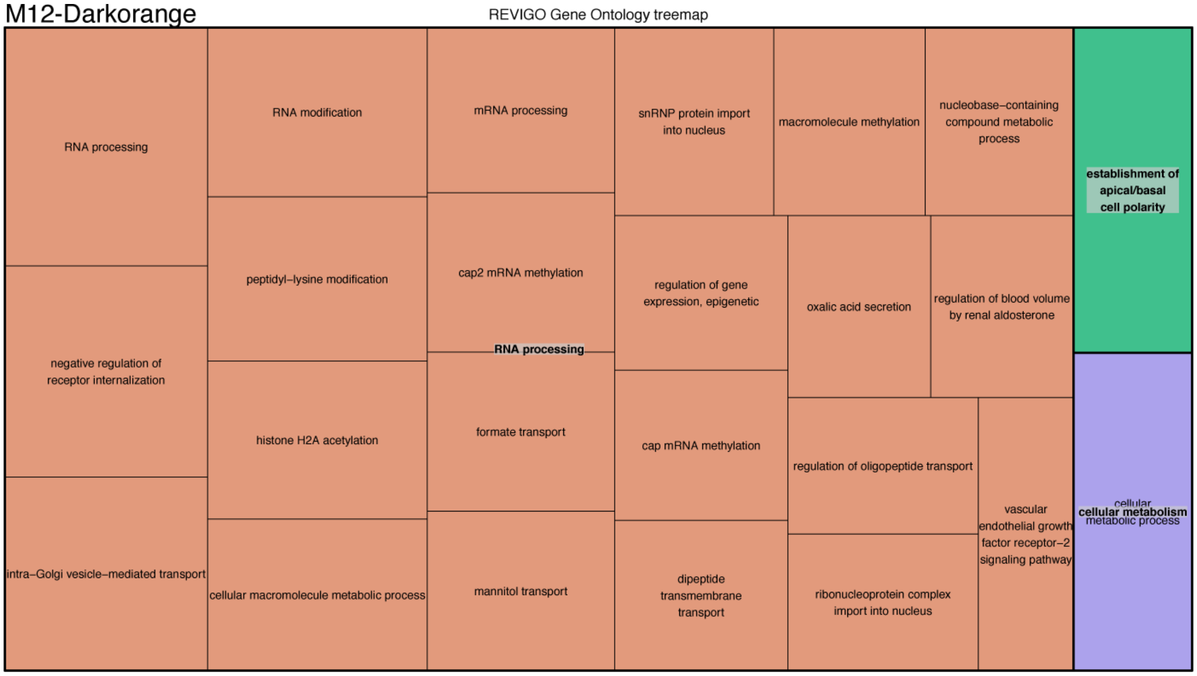

M3-Darkred

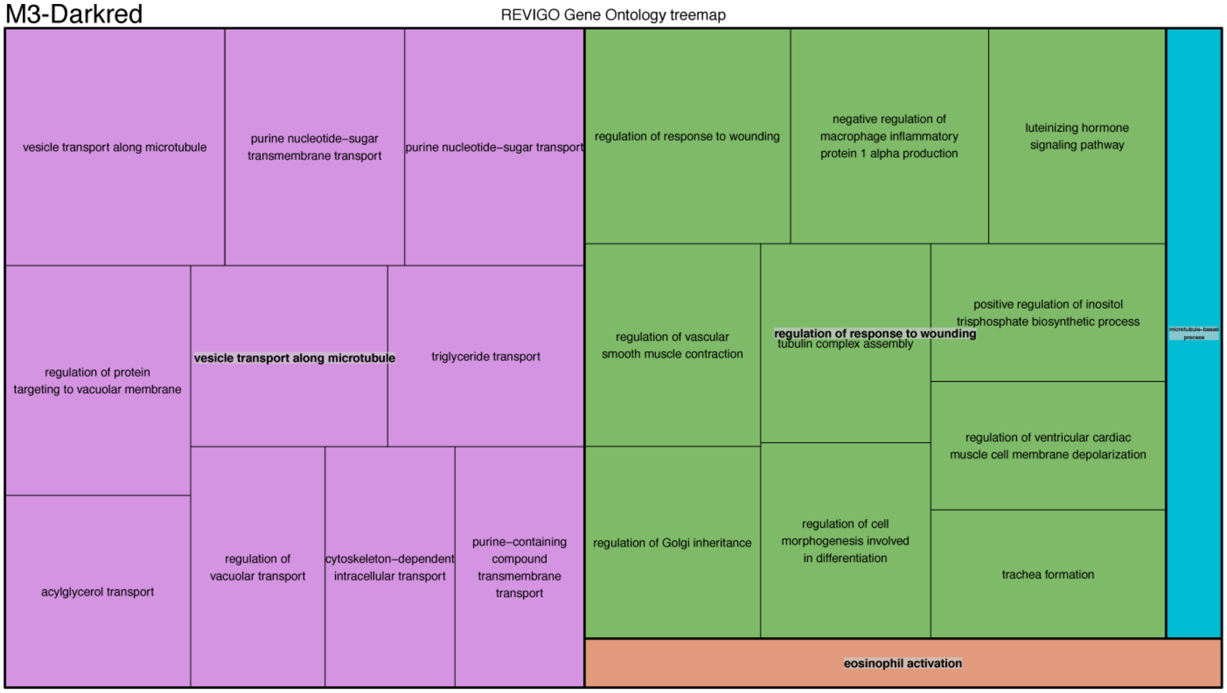

M8-Darkturquoise

REVIGO Gene Ontology treemap

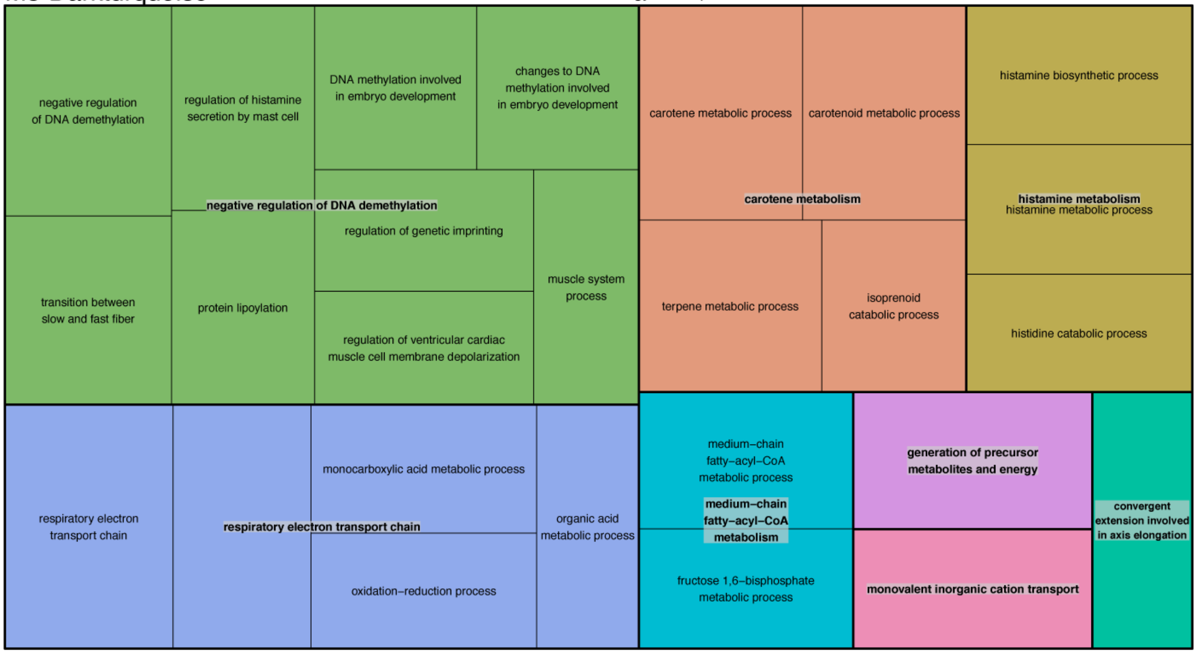

M1-Green

REVIGO Gene Ontology treemap

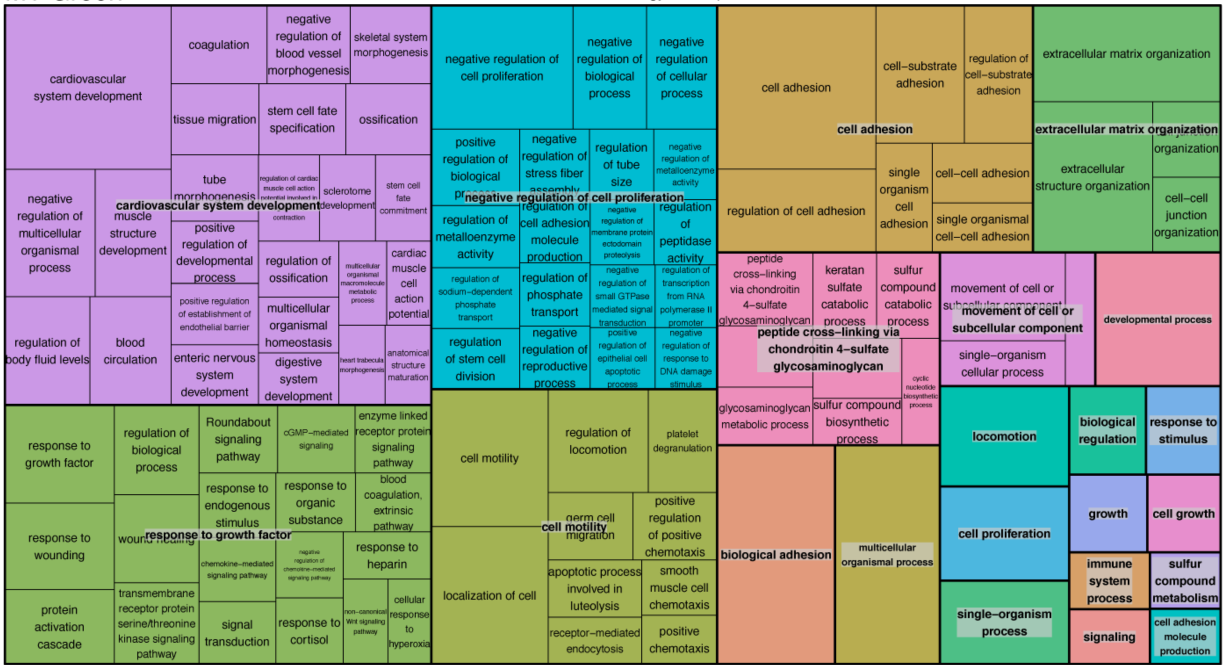

M16-Lightyellow

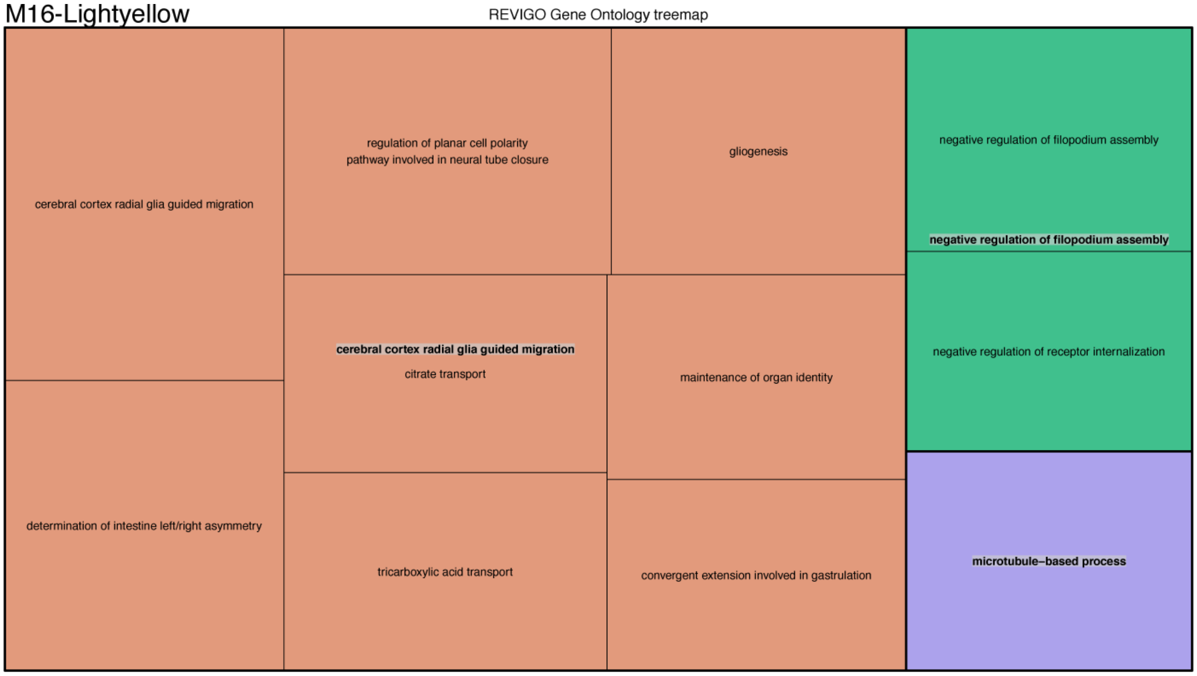

M17-Orange

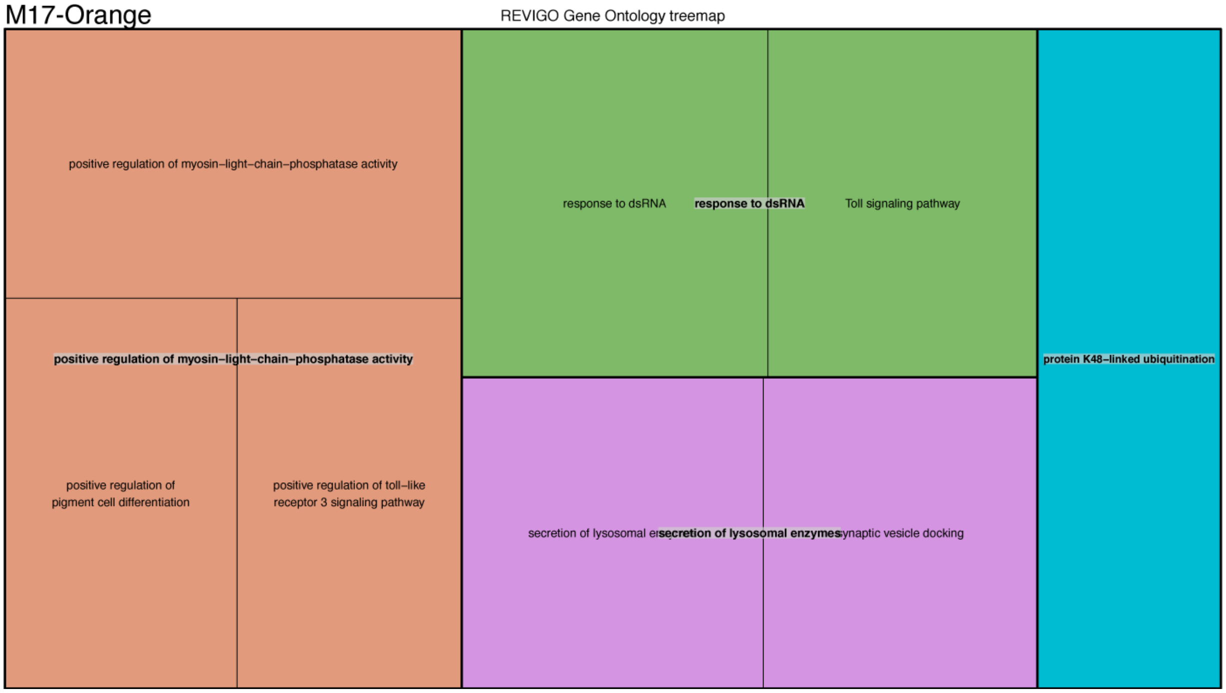

M6-Purple

REVIGO Gene Ontology treemap

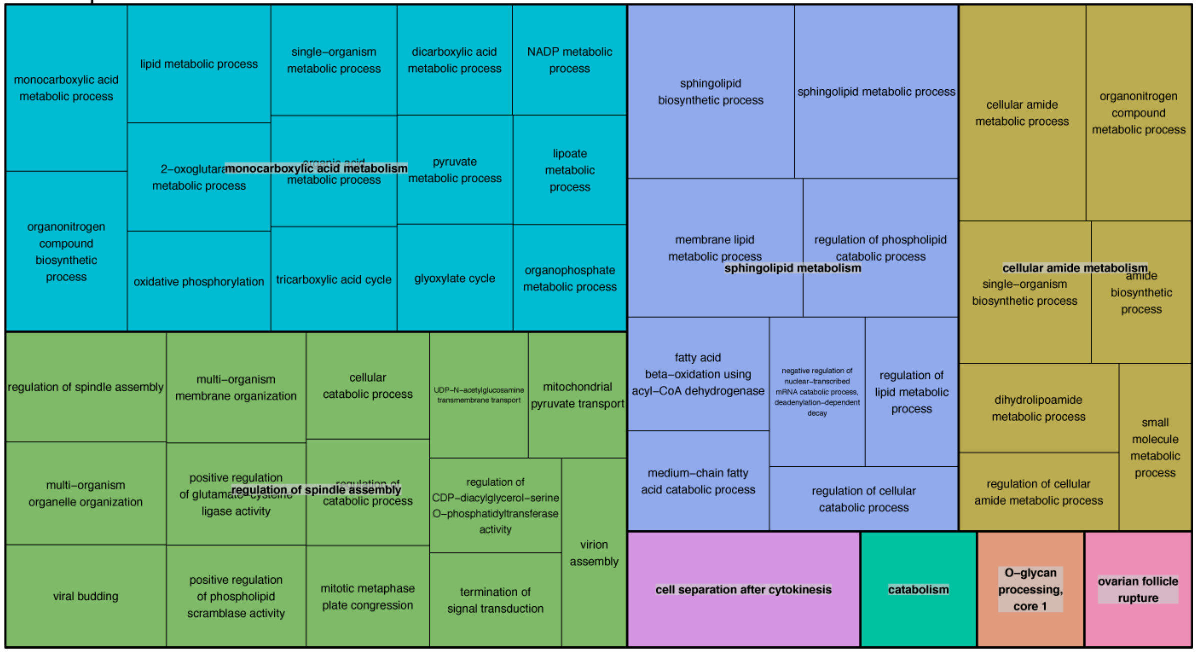

M15-Royalblue

REVIGO Gene Ontology treemap

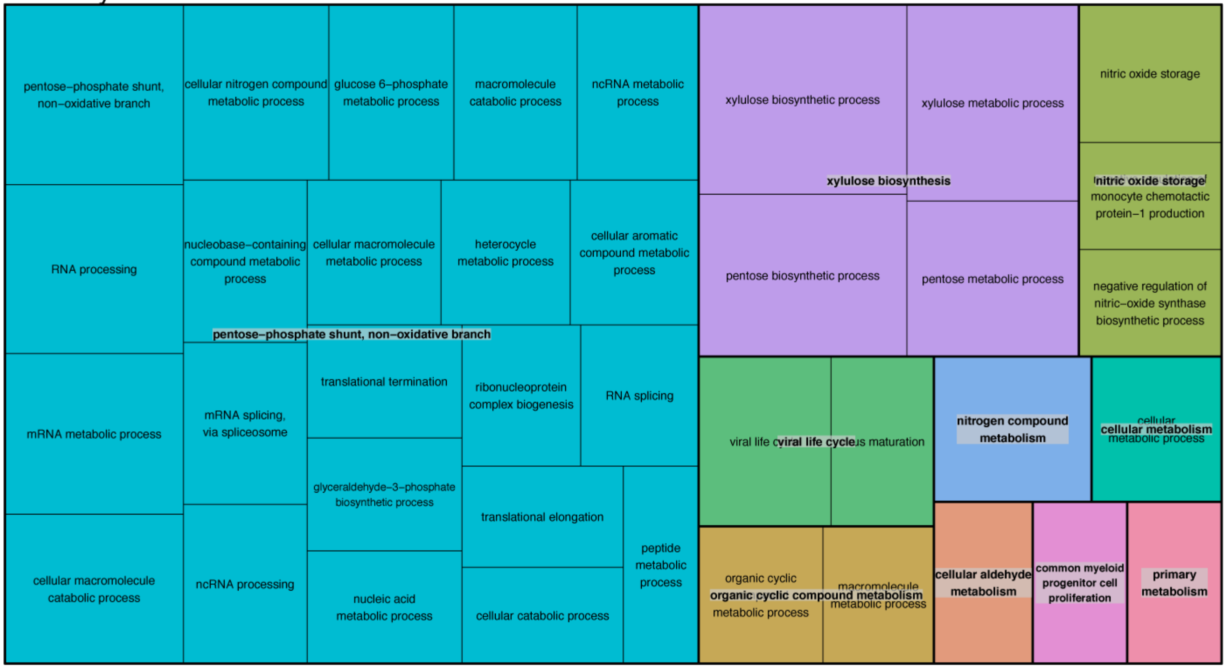

M14-Salmon

REVIGO Gene Ontology treemap

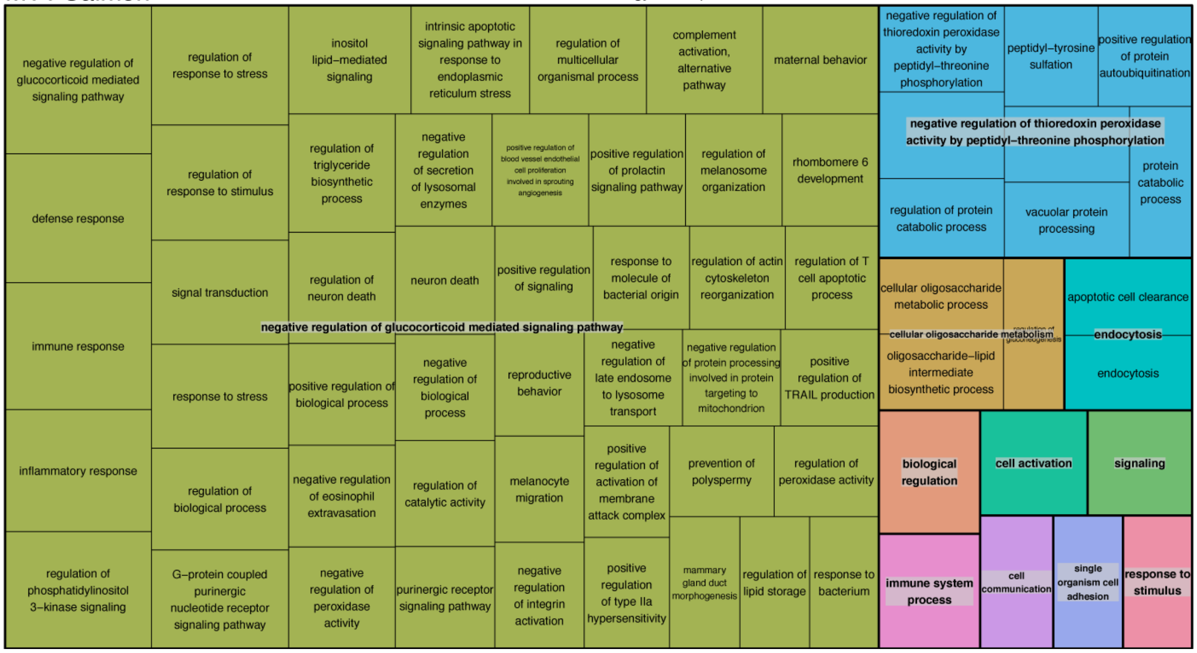

M4-Turquoise

REVIGO Gene Ontology treemap

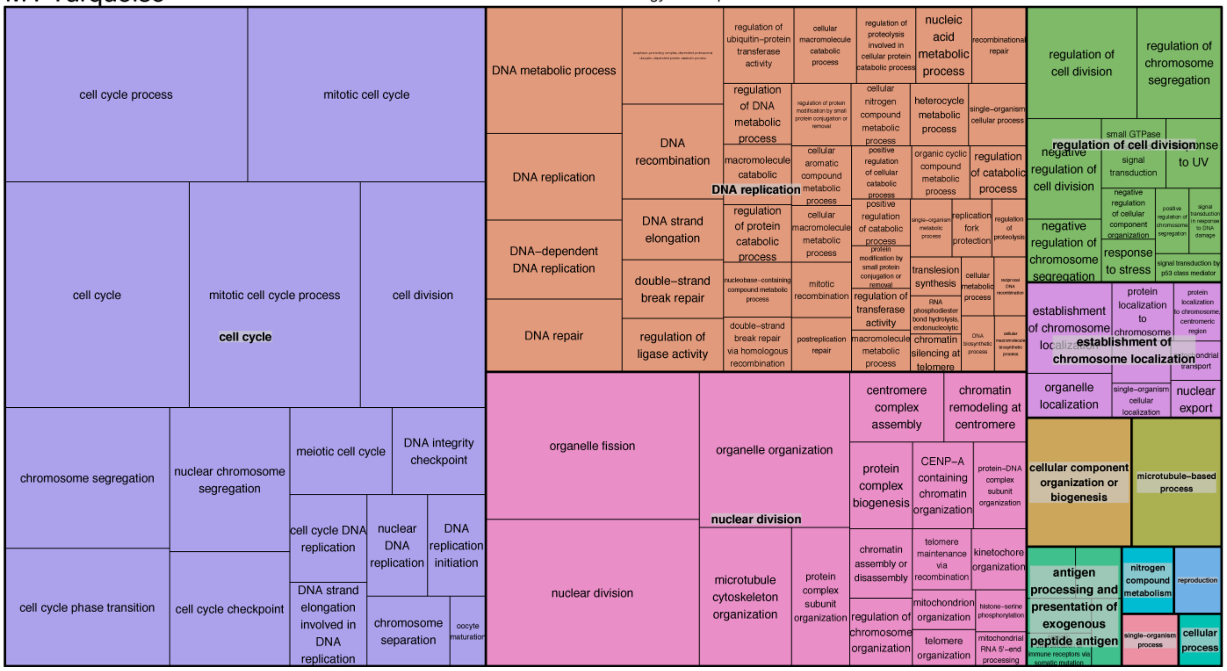

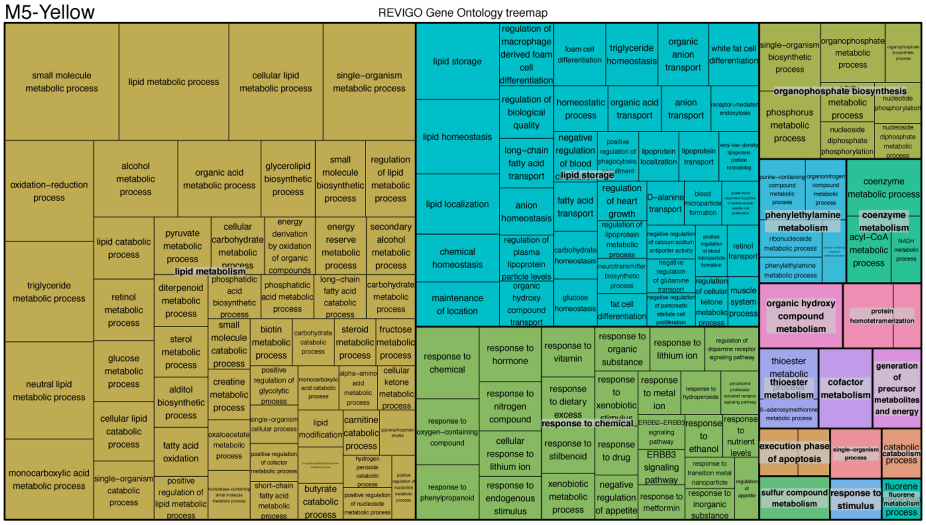

Supplementary Figure 3: ReviGO visualization of significantly enriched gene ontology terms among genes of each module.

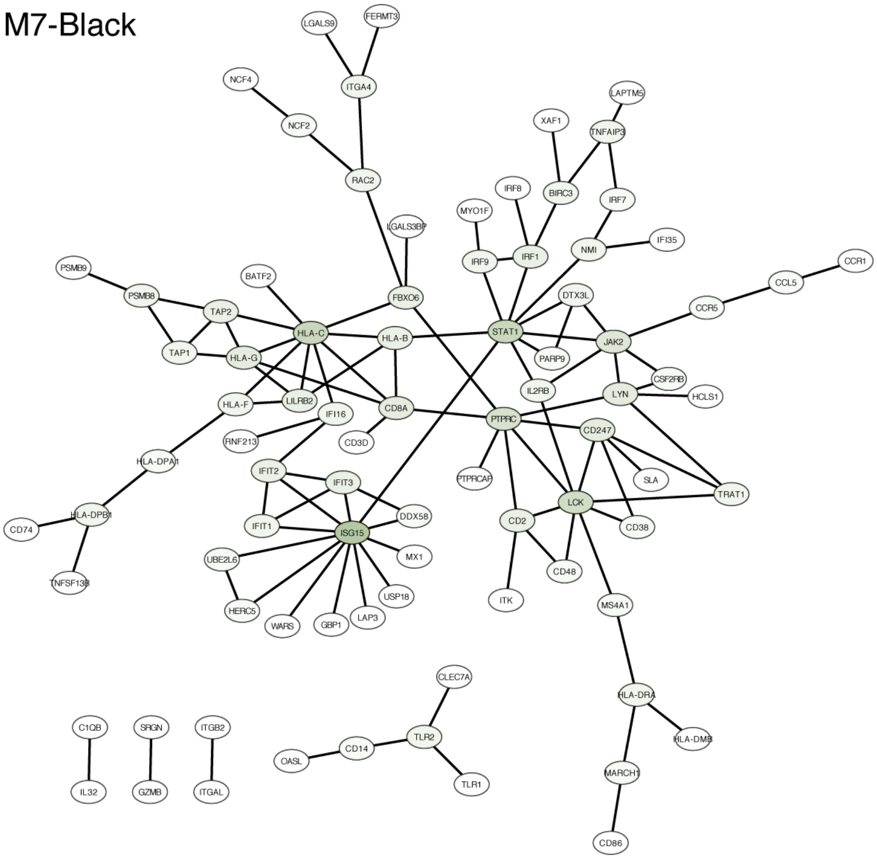
M7-Black
LGALS9
FERMT3
ITGA4
NCF4
NCF2
RAC2
LAPTM5
XAF1
TNFAIP3
IRF8
BIRC3
IRF7
MYO1F
LGALS3BP
IRF9
IRF1
NMI
IFI35
CCR1
PSMB9
BATF2
PSMB8
TAP2
FBXO6
DTX3L
CCL5
CCR5
HLA-C
HLA-B
STAT1
JAK2
TAP1
HLA-G
PARP9
CSF2RB
IL2RB
LYN
HCLS1
HLA-F
LILRB2
CD8A
IFI16
PTPRC
CD247
RNF213
CD3D
HLA-DPA1
IFIT2
IFIT3
SLA
PTPRCAP
TRAT1
LCK
CD2
DDX58
CD38
CD74
HLA-DPB1
IFIT1
ISG15
MX1
UBE2L6
CD48
TNFSF13B
USP18
ITK
MS4A1
HERC5
LAP3
GBP1
WARS
CLEC7A
HLA-DRA
C1QB
SRGN
ITGB2
TLR2
HLA-DMB
CD14
OASL
TLR1
MARCH1
IL32
GZMB
ITGAL
CD86

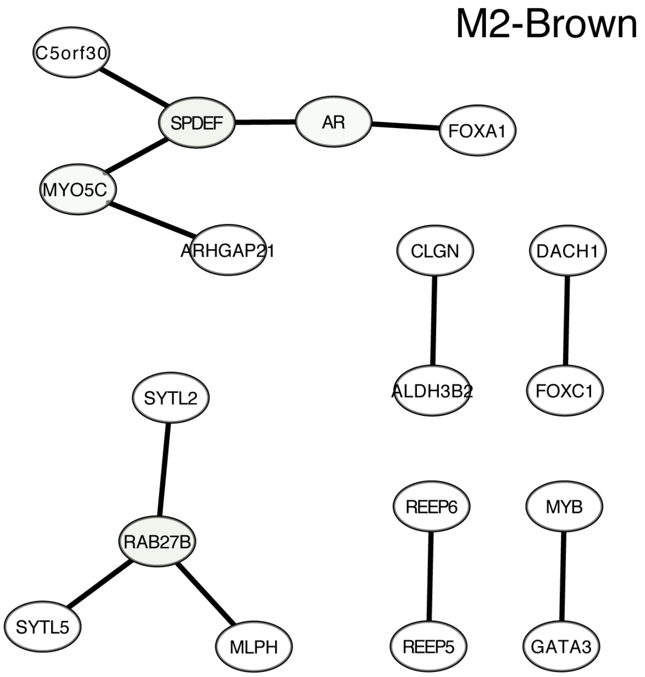
M2-Brown
C5orf30
SPDEF
AR
FOXA1
MYO5C
ARHGAP21
CLGN
DACH1
ALDH3B2
FOXC1
SYTL2
RAB27B
SYTL5
MLPH
REEP6
MYB
REEP5
GATA3

M9-Darkgrey

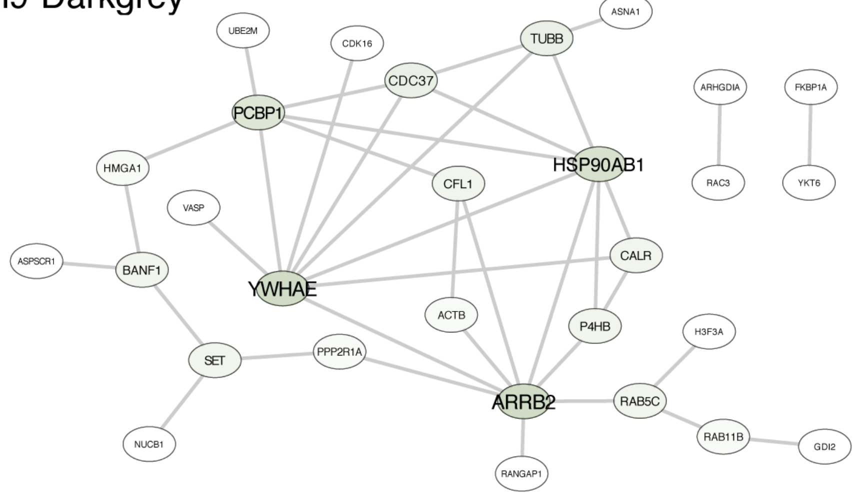

M12-Darkorange

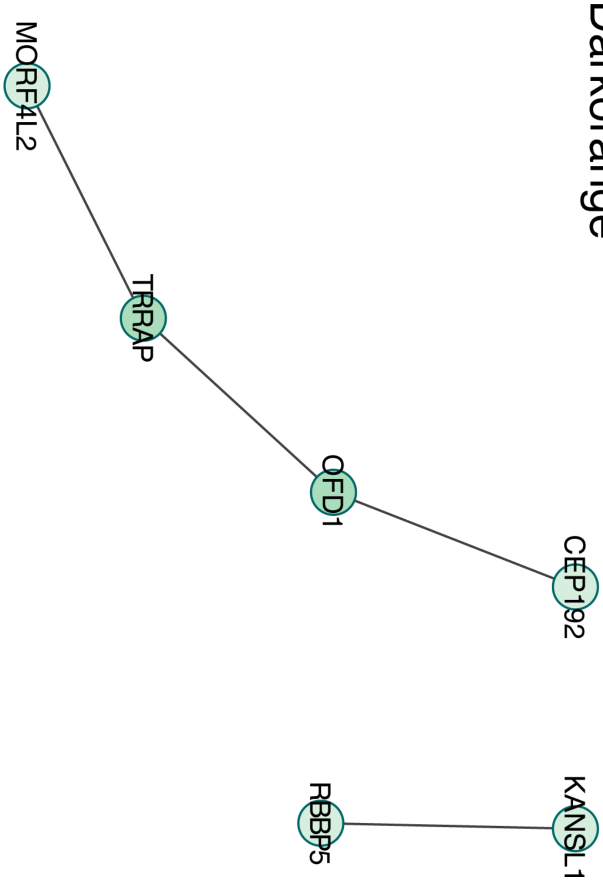

M8-darkturquoise

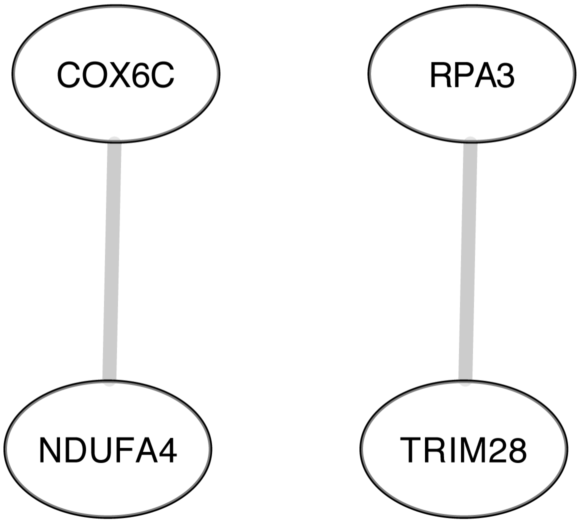

M1-green

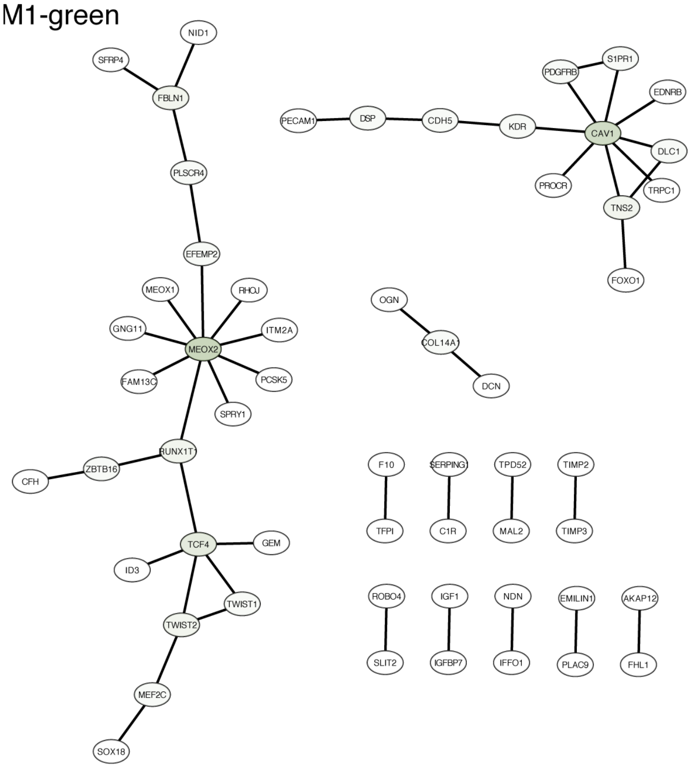

M6-Purple

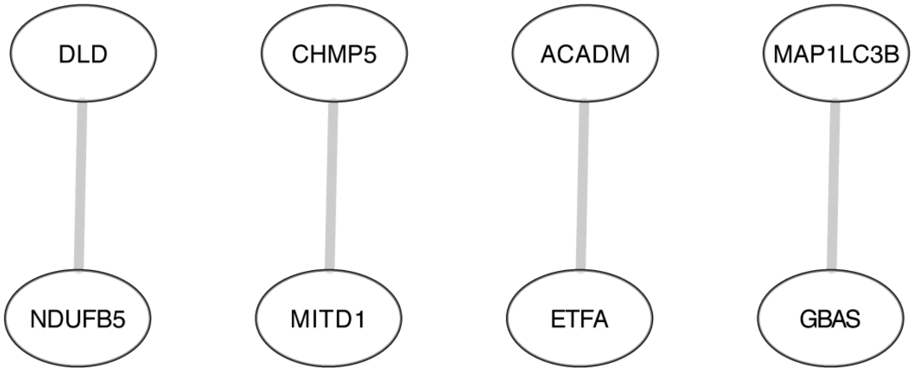

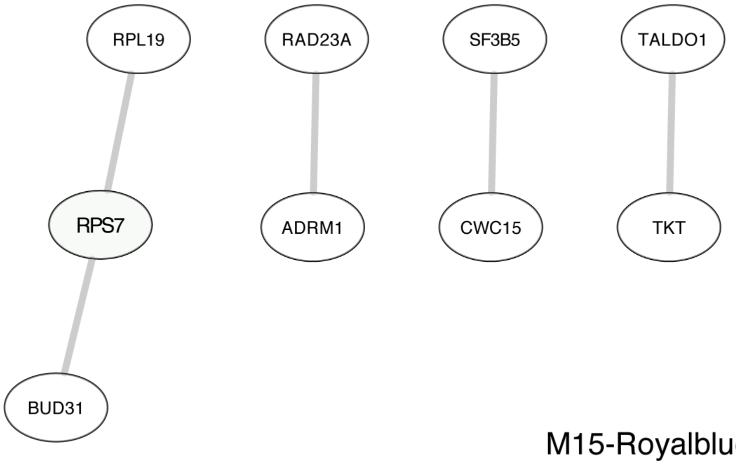

M15-Royalblue

M14-Salmon

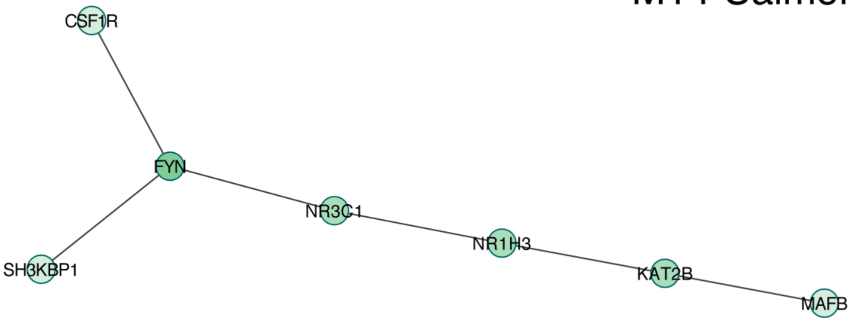

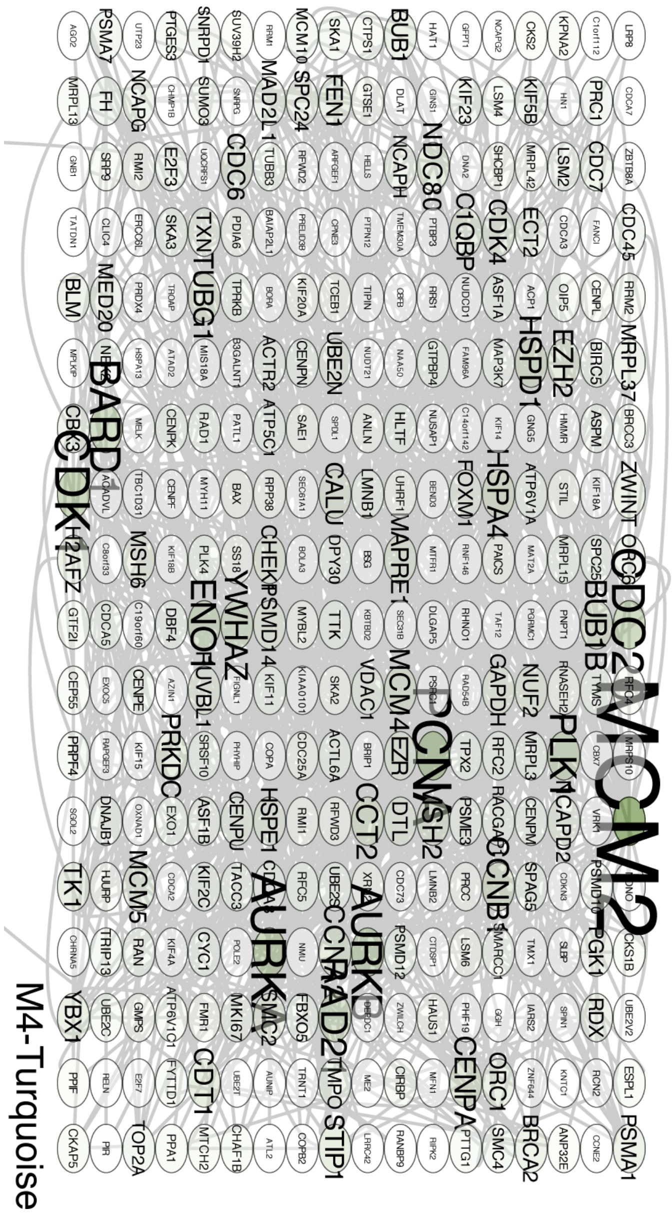

M5-Yellow

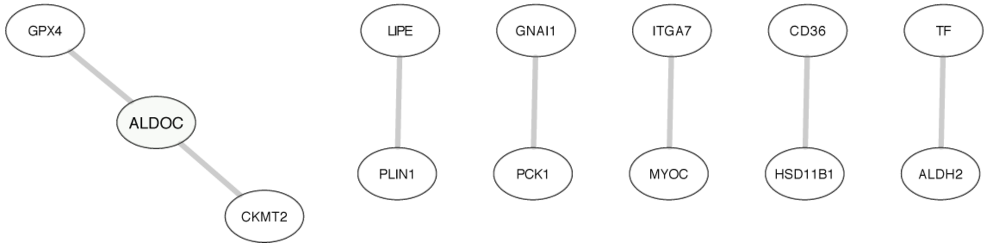

Supplementary Figure 4: Protein-protein interaction network for genes in each module. Green color shade indicates node degree.

BLACK

Best cutoff | Median

Weighted

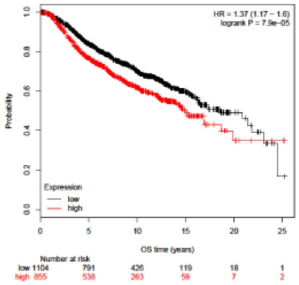

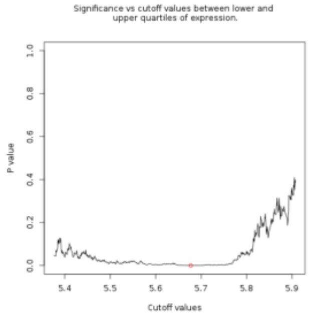

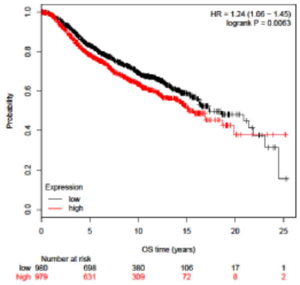

Not weighted

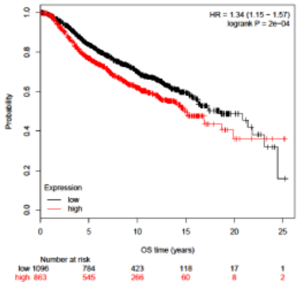

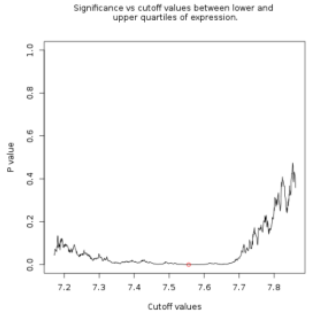

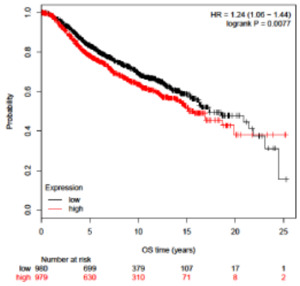

BROWN

Best cutoff | Median

Weighted

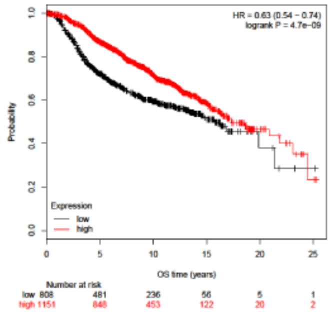

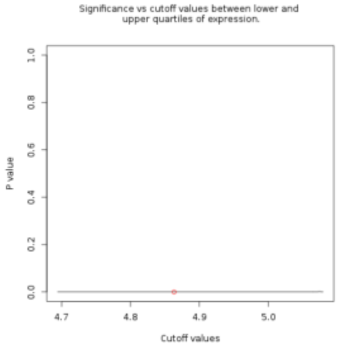

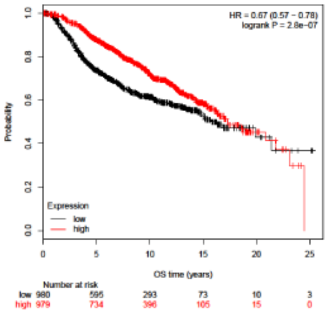

Not weighted

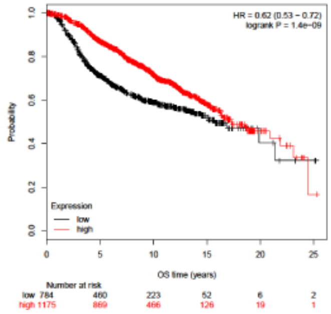

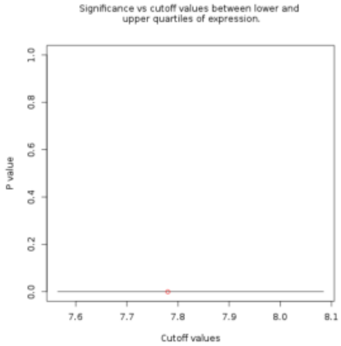

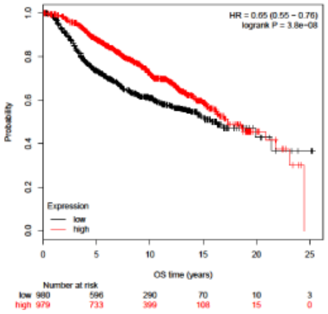

Cyan

Best cutoff | Median

Weighted / Not weighted

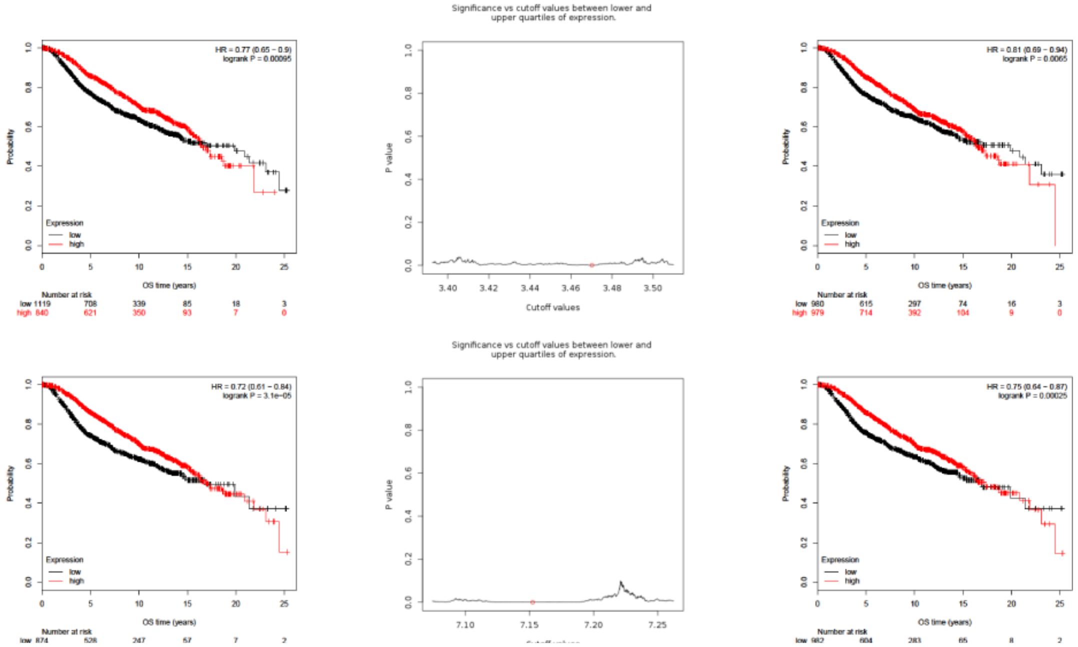

Darkgrey

Best cutoff | Median

Weighted / Not weighted

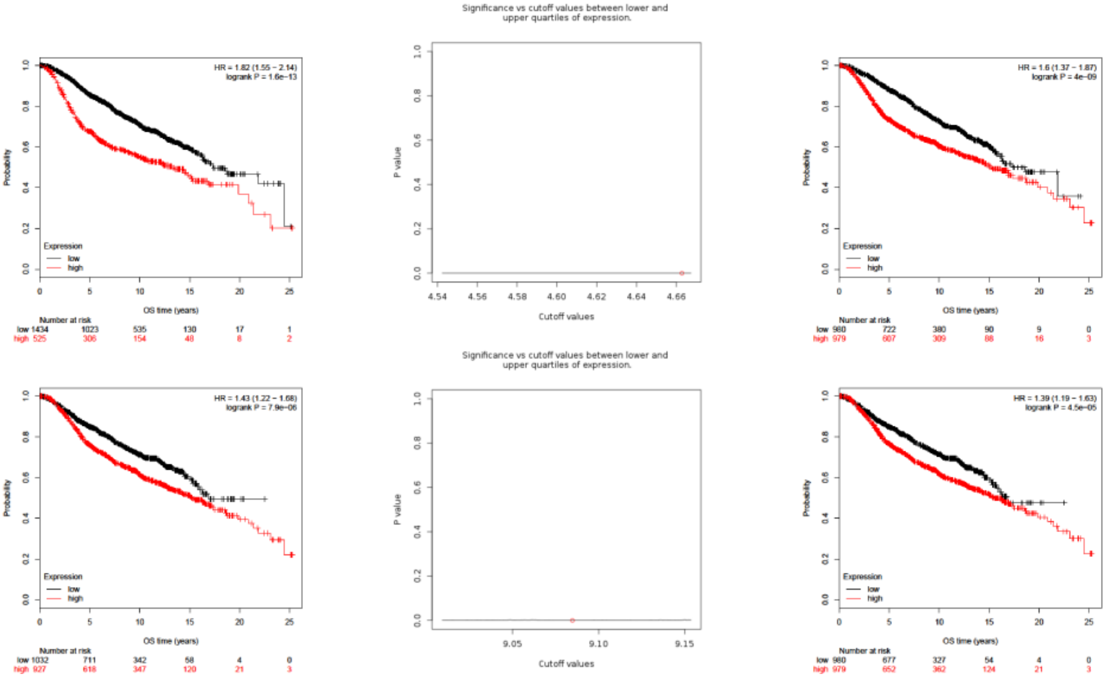

DARKORANGE

Best cutoff | Median

Weighted / Not weighted

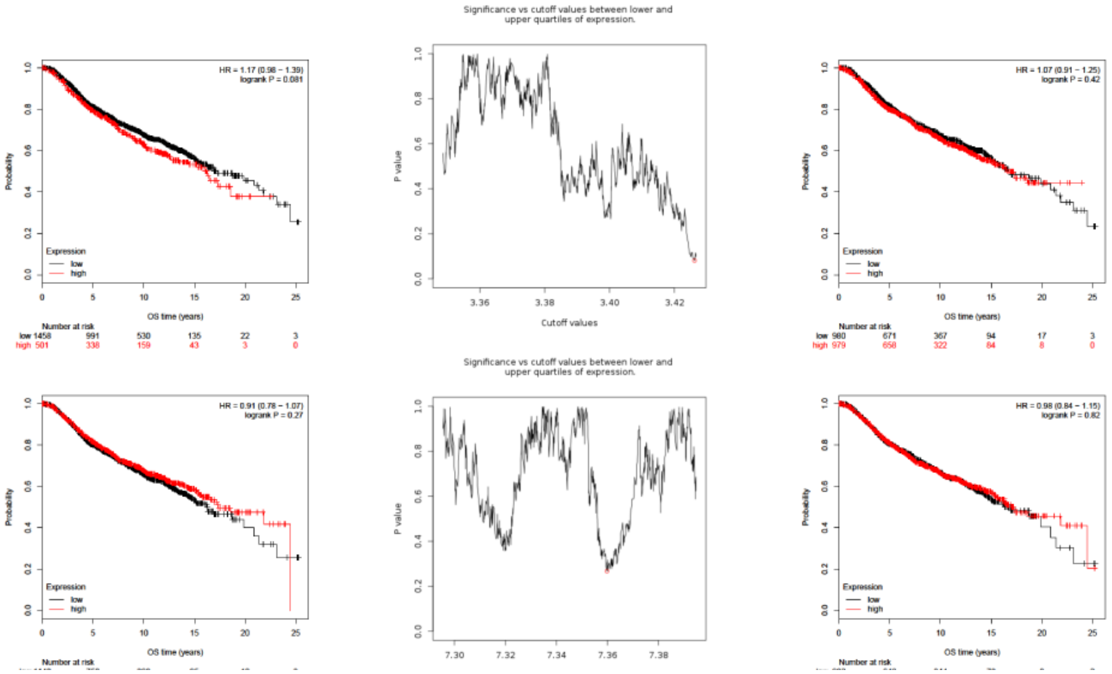

DARKRED

Best cutoff | Median

Weighted / Not weighted

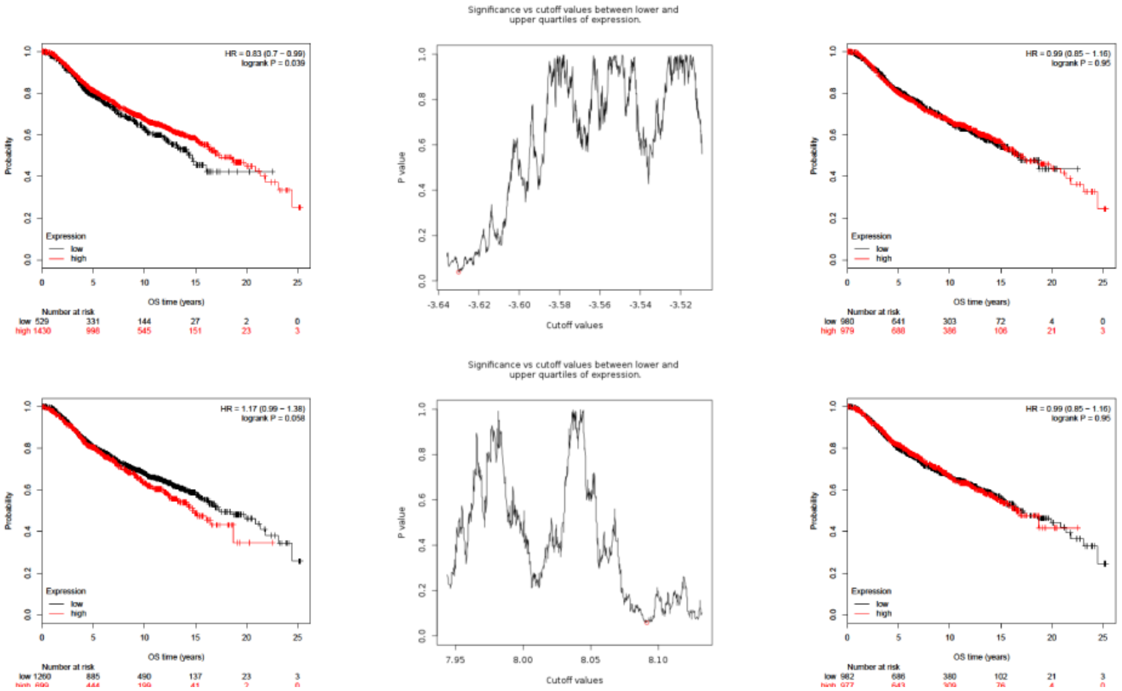

DARKTURQUOISE

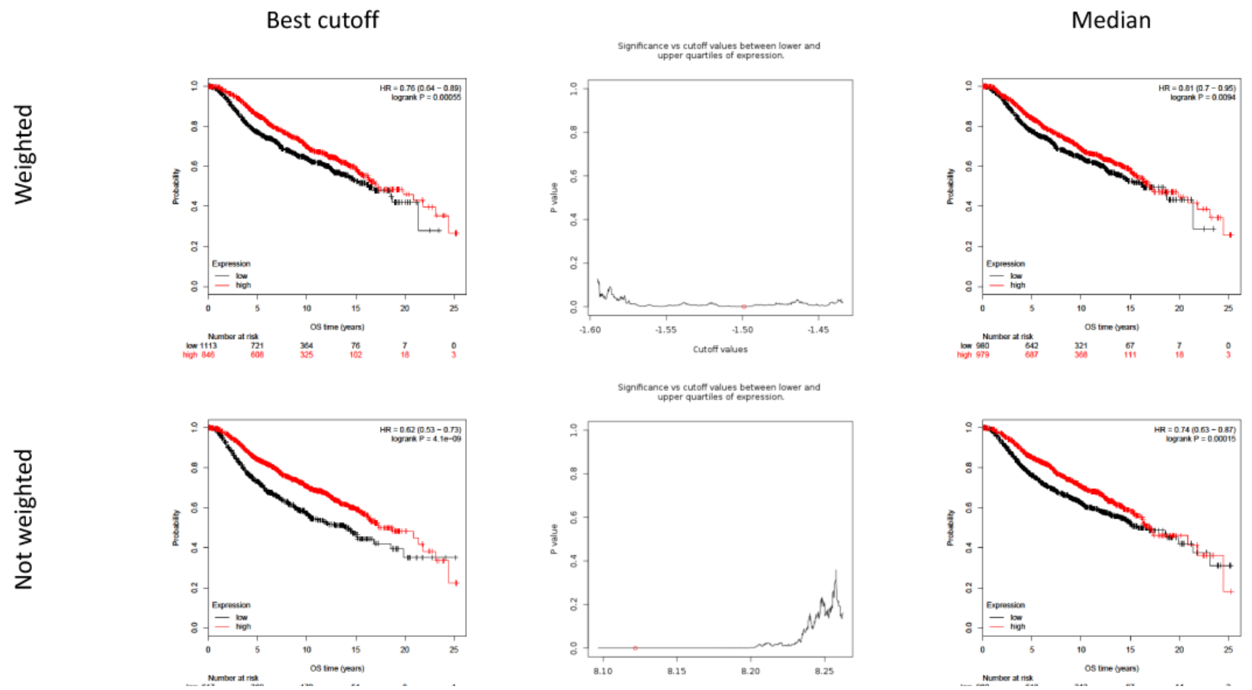

GREEN

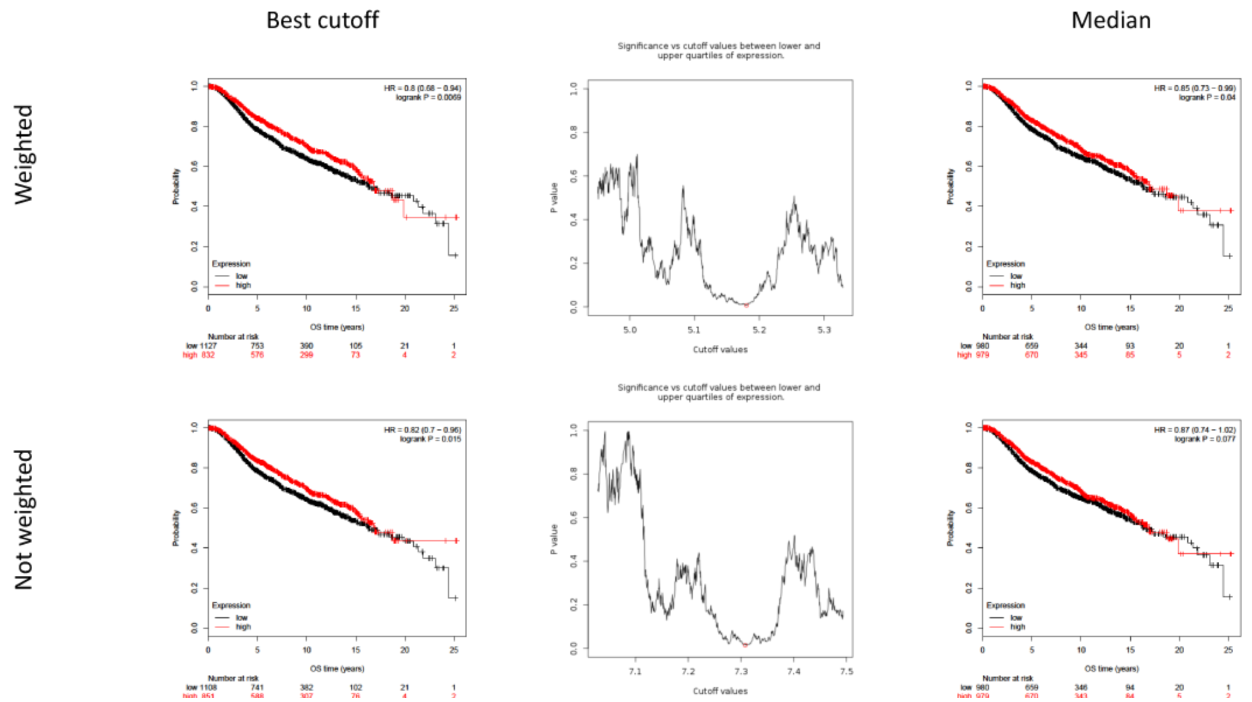

GREENYELLOW

Best cutoff | Median

Weighted

Not weighted

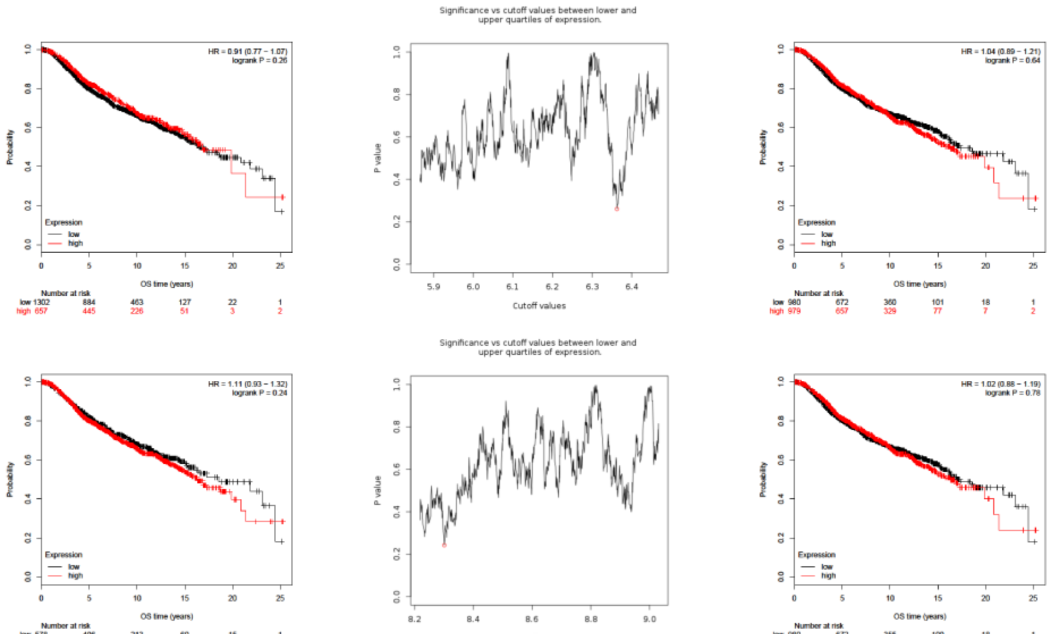

GREY60

Best cutoff | Median

Weighted

Not weighted

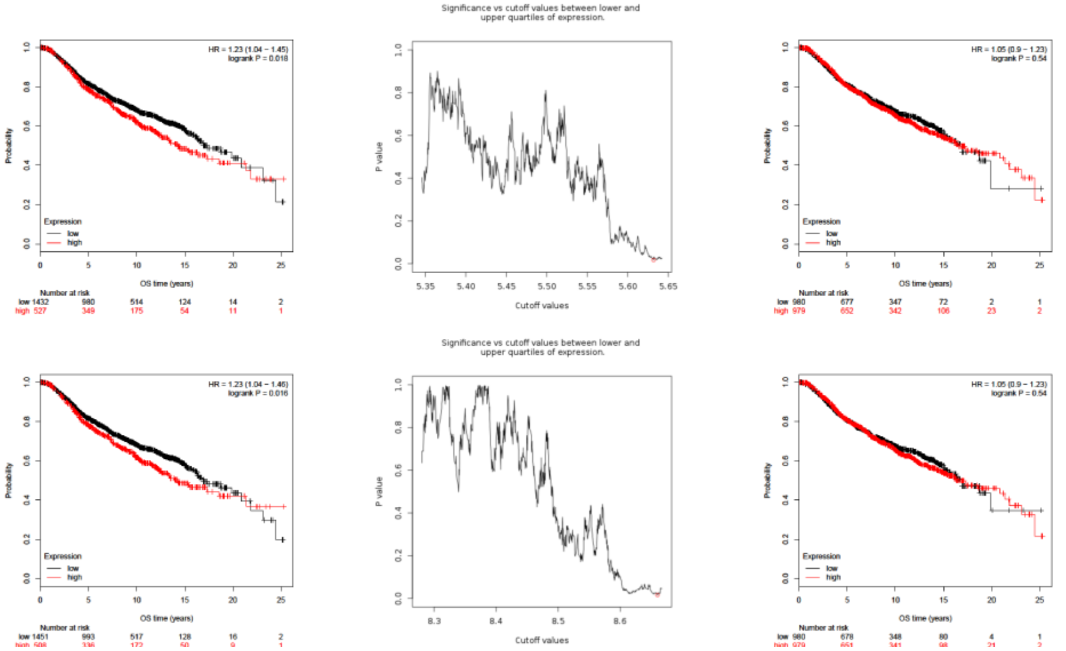

GREY500

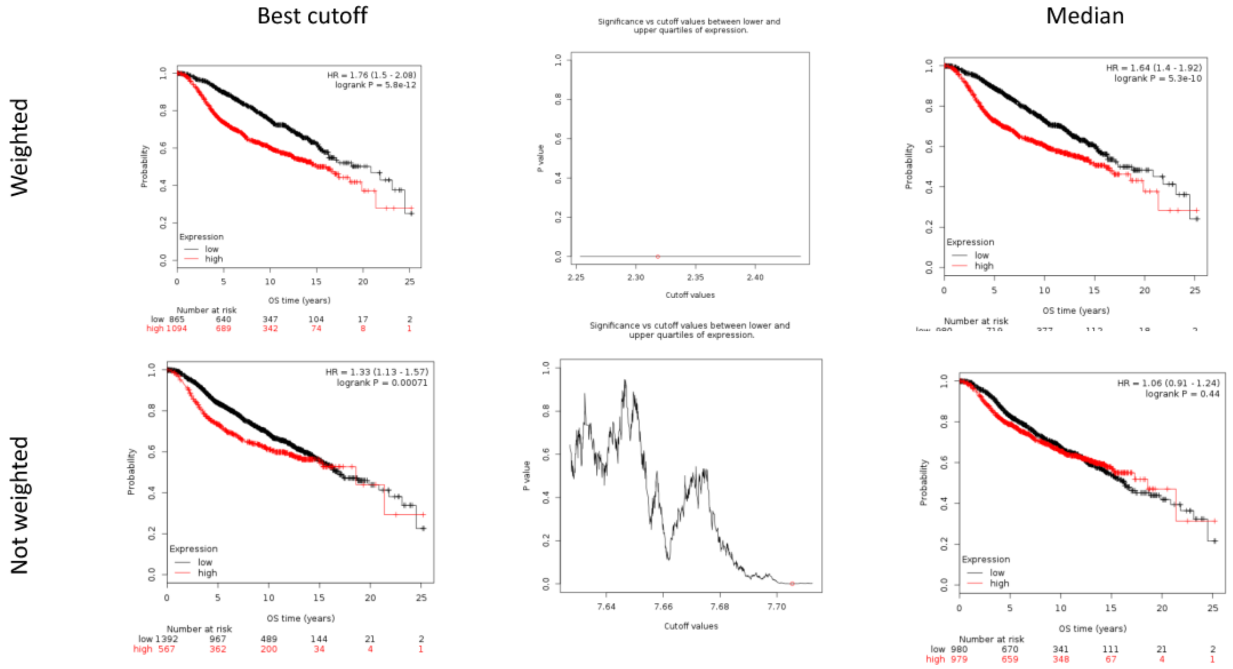

LIGHTYELLOW

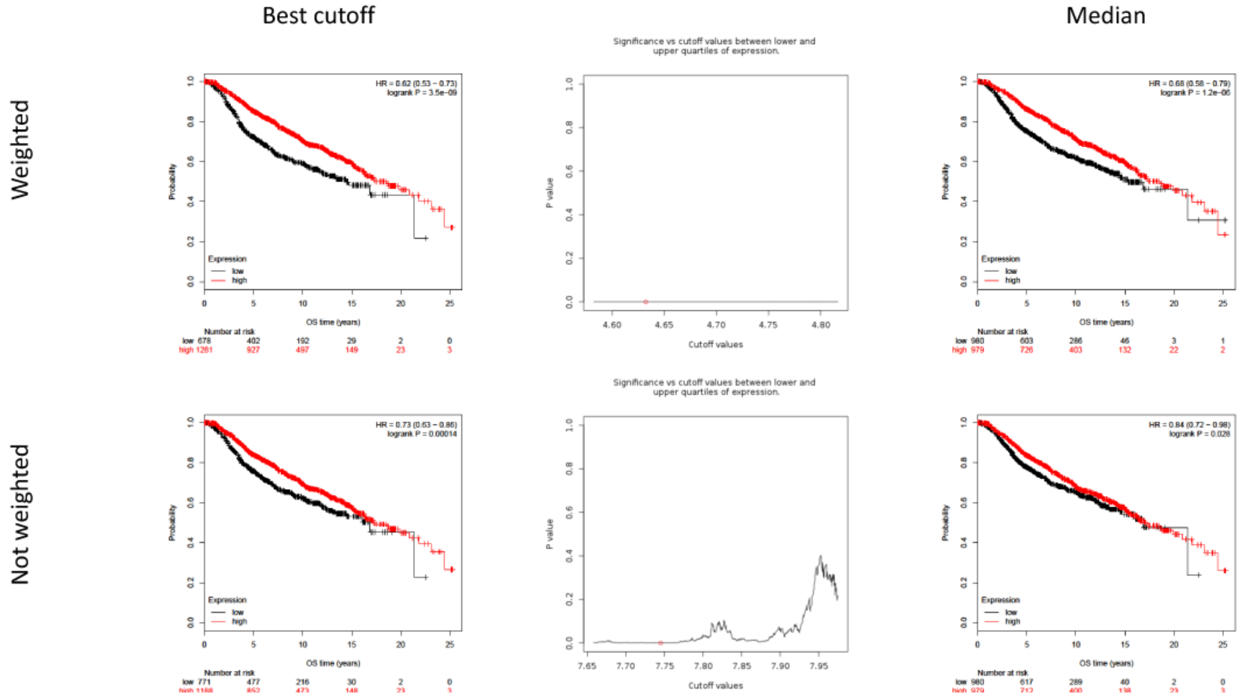

ORANGE

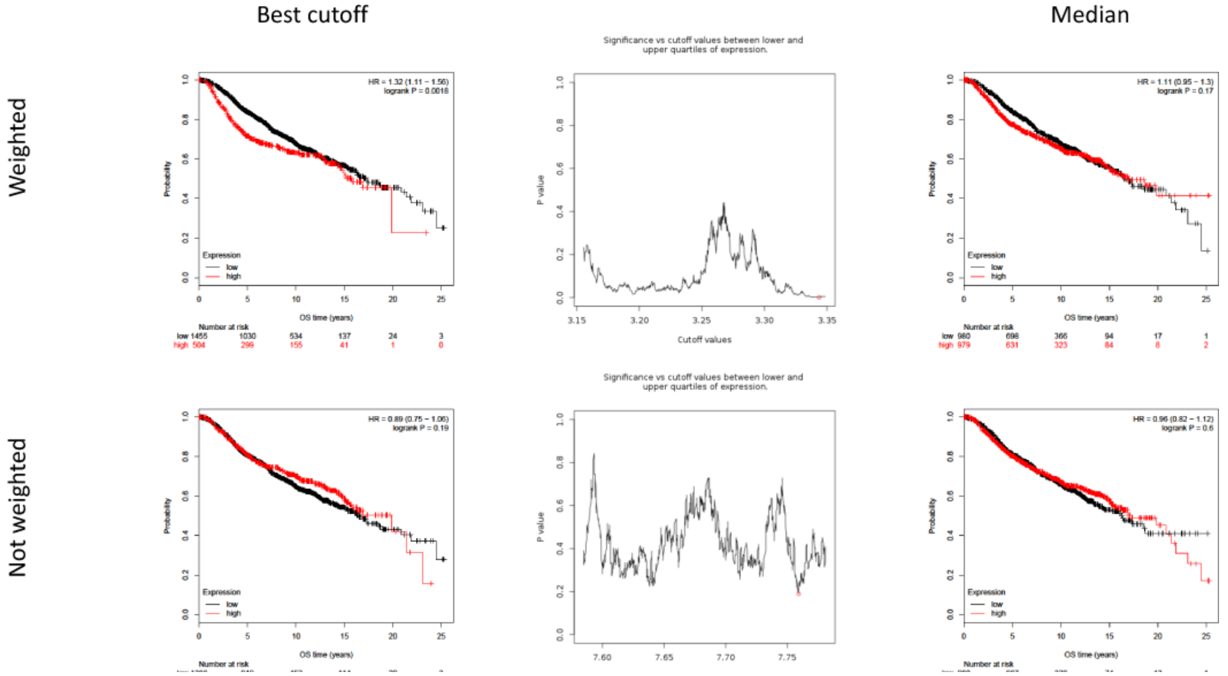

PURPLE

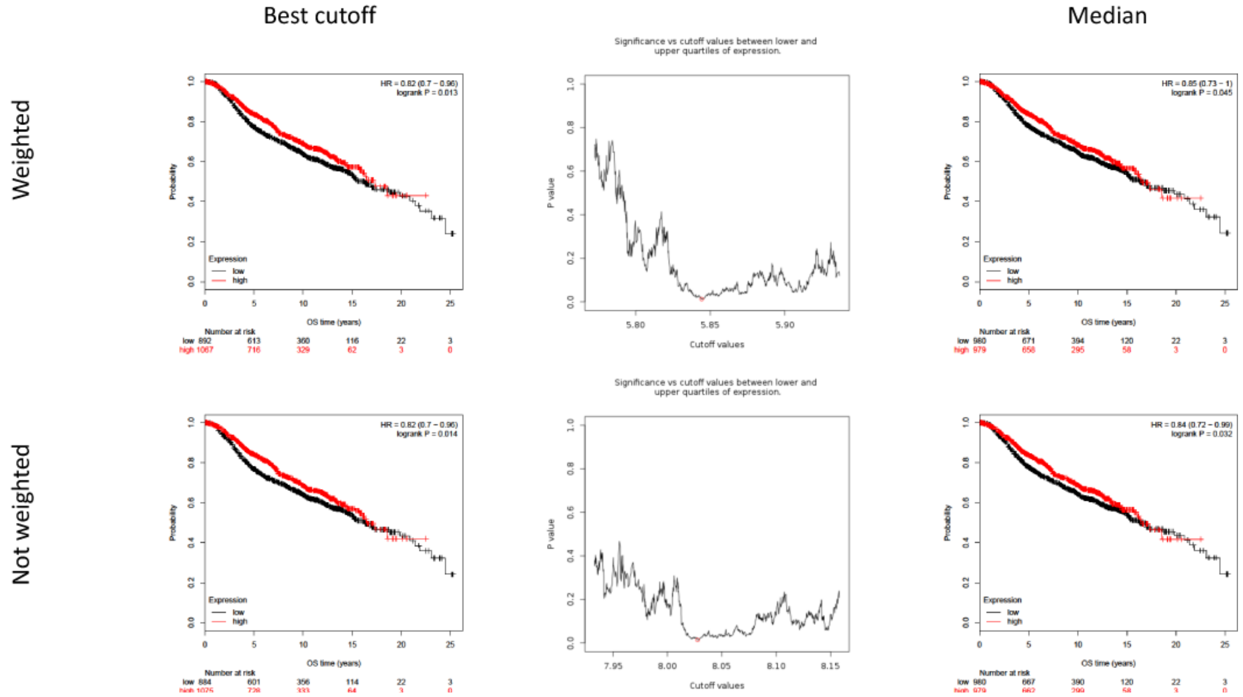

Royalblue

Best cutoff | Median

Weighted / Not weighted

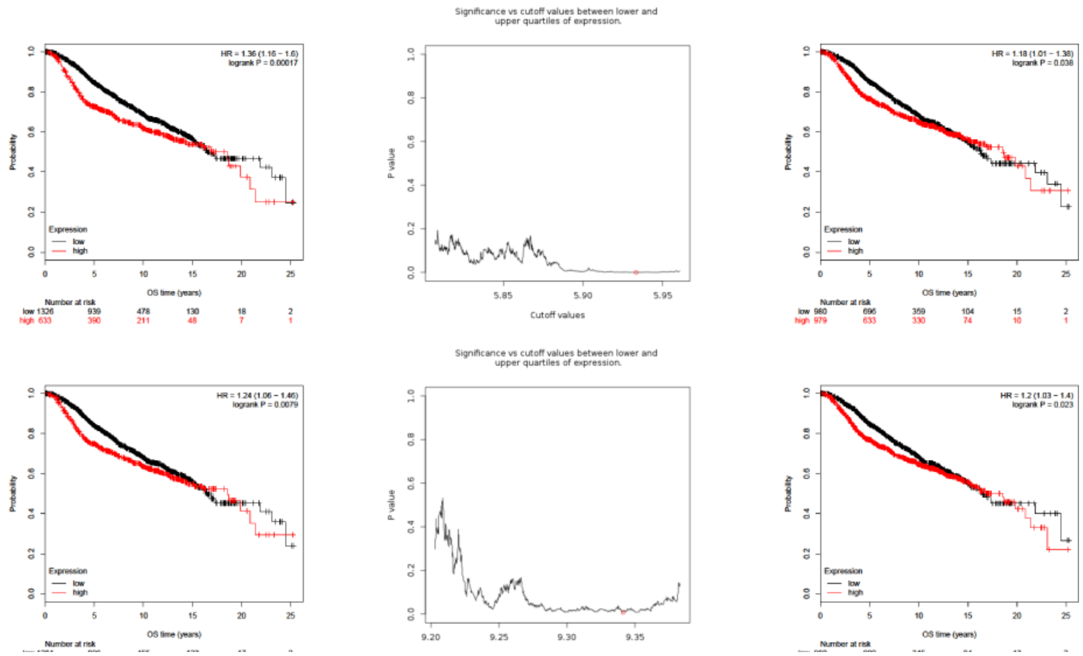

Salmon

Best cutoff | Median

Weighted / Not weighted

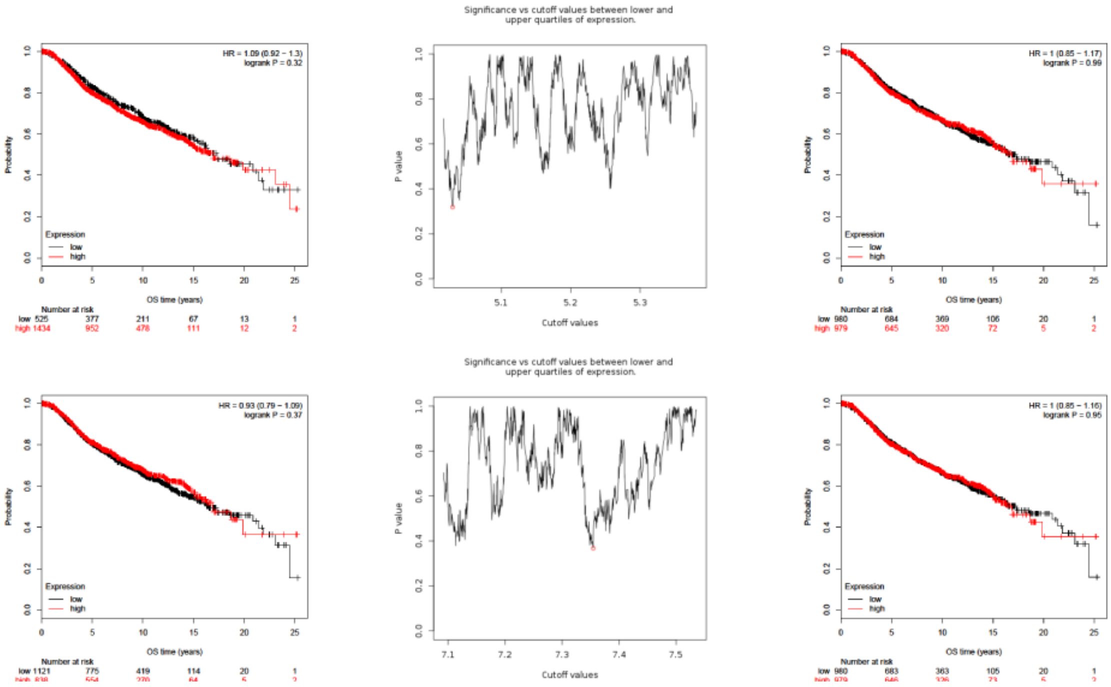

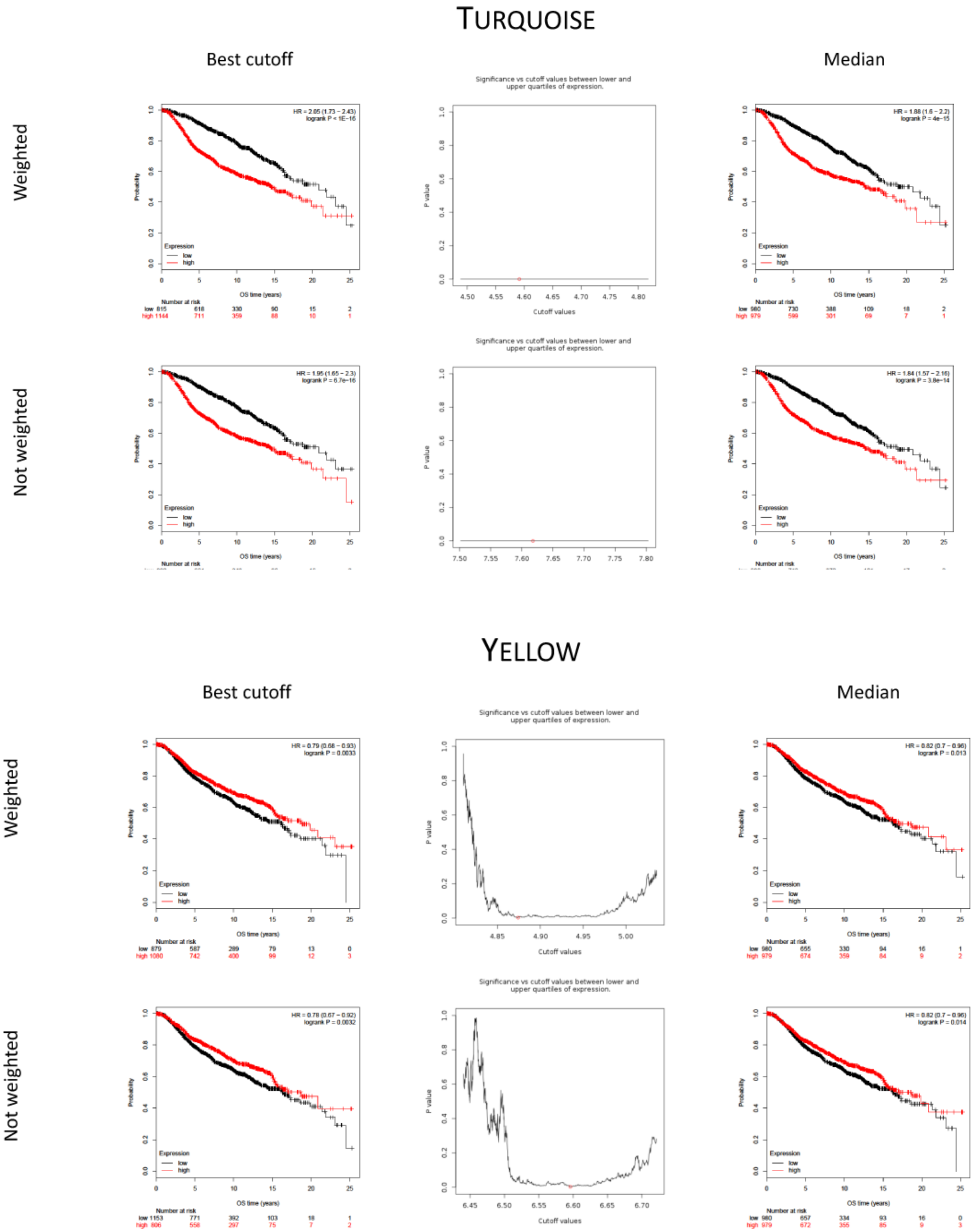

Supplementary Figure 5: KM plots for genes in modules splitting Metabric samples in low-expressing and high-expressing gene sets. Upper panels labeled show results using genes values weighted by gene membership in the corresponding module.

Supplementary Table 1: Overlaps between genes in modules and previously reported breast cancer dysregulated genes. *P*-values were obtained by means of a Fisher's exact test. See Supplementary_Table_1

Supplementary Table 2: Expression summary statistics of genes in modules in normal and tumor Metabric samples. Rows are colored according to the direction of changes (green, up-regulated and red, down-regulated)

sample	genelist	min	Q1	median	Q3	max	*p*_value
normal	black	9.846	10.095	10.183	10.32	11.005	
tumour	black	8.268	10.411	10.81	11.282	12.911	3.79E–42
normal	brown	9.584	10.014	10.266	10.501	11.183	
tumour	brown	9.079	10.753	11.144	11.438	12.322	8.81E–40
normal	cyan	10.059	10.178	10.215	10.262	10.373	
tumour	cyan	8.407	10.171	10.279	10.375	10.788	2.07E–08
normal	darkgrey	11.807	11.884	11.925	11.975	12.089	
tumour	darkgrey	10.443	12.951	13.04	13.129	13.577	1.12E–88
normal	darkorange	10.159	10.28	10.349	10.4	10.539	
tumour	darkorange	8.446	10.429	10.5	10.576	10.949	1.86E–55
normal	darkred	10.318	10.514	10.576	10.654	10.87	
tumour	darkred	8.932	10.819	10.917	11.024	11.62	2.75E–68
normal	darkturquoise	11.099	11.225	11.272	11.33	11.617	
tumour	darkturquoise	9.559	11.72	11.837	11.959	12.478	1.26E–86
normal	green	10.128	10.943	11.194	11.363	11.642	
tumour	green	8.479	10.106	10.378	10.661	12.027	6.26E–62
normal	greenyellow	10.243	10.789	10.964	11.168	12.182	
tumour	greenyellow	9.617	11.485	12.02	12.531	14.175	3.66E–56
normal	grey	10.283	10.396	10.432	10.452	10.49	
tumour	grey	8.595	10.484	10.536	10.589	10.768	4.88E–59
normal	grey60	9.086	9.883	10.346	10.553	10.917	
tumour	grey60	8.749	10.877	11.106	11.288	11.892	2.20E–69
normal	lightyellow	10.555	10.728	10.808	10.898	11.209	
tumour	lightyellow	8.488	10.551	10.705	10.879	11.619	2.85E–09
normal	orange	10.136	10.512	10.587	10.657	10.807	
tumour	orange	8.364	10.367	10.484	10.621	11.156	5.00E–11
normal	purple	10.562	10.799	10.889	11.007	11.238	
tumour	purple	9.37	11.261	11.428	11.573	12.624	2.66E–68
normal	royalblue	11.33	11.428	11.48	11.541	11.749	
tumour	royalblue	10.078	12.233	12.335	12.445	13.087	1.12E–88
normal	salmon	9.905	10.347	10.498	10.7	11.221	
tumour	salmon	8.219	10.237	10.468	10.722	11.846	0.103305466
normal	turquoise	10.261	10.325	10.35	10.401	10.914	
tumour	turquoise	8.941	10.856	11.049	11.26	12.003	1.36E–86
normal	yellow	9.846	10.454	11.16	11.874	12.84	
tumour	yellow	7.864	9.607	9.724	9.926	13.217	1.07E–76

Supplementary Table 3: Gene Ontology categories enriched in each gene module. See Supplementary_Table_3

Supplementary Table 4: List of drugs associated to hub genes in modules. See Supplementary_Table_4

Supplementary Table 5: Achieved hazard rate and *p*-values for each module

	weighted						unweighted					
	HR		*p*_value		cutoff		HR		*p*_value		cutoff	
	best_ cutoff	median	best_ cutoff	median	best_ cutoff	median	best_ cutoff	median	best_ cutoff	median	best_ cutoff	median
M7-black	1.37	1.24	0.000079	0.0063	5.68	5.62	1.34	1.24	0.0002	0.0077	7.56	7.49
M2-brown	0.63	0.67	4.7E–09	0.00000028	4.86	4.93	0.62	0.65	1.4E–09	0.000000038	7.78	7.88
M10-cyan	0.77	0.81	0.00095	0.0065	3.47	3.46	0.72	0.75	0.000031	0.00025	7.15	7.17
M9-darkgrey	1.82	1.6	1.6E–13	0.000000004	4.66	4.6	1.43	1.39	0.0000079	0.000045	9.08	9.08
M12-darkorange	1.17	1.07	0.081	0.42	3.43	3.39	0.91	0.98	0.27	0.82	7.36	7.34
M3-darkred	0.83	0.99	0.039	0.95	–3.63	–3.57	1.17	0.99	0.058	0.95	8.09	8.04
M8-darkturquoise	0.76	0.81	0.00055	0.0094	–1.5	–1.52	0.62	0.74	4.1E–09	0.00015	8.12	8.18
M1-green	0.8	0.85	0.0069	0.04	5.18	5.13	0.82	0.87	0.015	0.077	7.31	7.26
M11-greenyellow	0.91	1.04	0.26	0.64	6.36	6.18	1.11	1.02	0.24	0.78	8.3	8.64
M0-grey	1.76	1.64	5.80E–12	5.30E–10	2.32	2.34	1.33	1.06	0.00071	0.44	7.71	7.67
M13-grey60	1.23	1.05	0.018	0.54	5.63	5.51	1.23	1.05	0.016	0.54	8.66	8.49
M16-lightyellow	0.62	0.68	3.5E–09	0.0000012	4.63	4.7	0.73	0.84	0.00014	0.028	7.75	7.8
M17-orange	1.32	1.11	0.0018	0.17	3.34	3.25	0.89	0.96	0.19	0.6	7.76	7.68
M6-purple	0.82	0.85	0.013	0.045	5.84	5.86	0.82	0.84	0.014	0.032	8.03	8.05
M15-royalblue	1.36	1.18	0.00017	0.038	5.93	5.88	1.24	1.2	0.0079	0.023	9.34	9.29
M14-salmon	1.09	1	0.32	0.99	5.03	5.18	0.93	1	0.37	0.95	7.35	7.29
M4-turquoise	2.05	1.88	1E–16	4E–15	4.59	4.64	1.95	1.84	6.7E–16	3.8E–14	7.62	7.64
M5-yellow	0.79	0.82	0.0033	0.013	4.87	4.89	0.78	0.82	0.0032	0.014	6.6	6.54
