# Supplementary material for: Transcriptome evolution from breast epithelial cells to basal-like tumors
Source: Oncotarget. 2017 Dec 8;9(1):453–63. doi: 10.18632/oncotarget.23065 (PMC5787480; doi:10.18632/oncotarget.23065)
Supplement: Supplementary file 3 [file oncotarget-09-453-s003.pdf]

GOBPID	Pvalue	OddsRatio	ExpCount	Count	Size	Term
GO:0044281	1.66E-18	6.322353029	15.91670015	54	2377	small molecule metabolic process
GO:0006629	8.29E-18	7.522473971	8.042051694	39	1201	lipid metabolic process
GO:0044255	2.23E-15	7.453027976	6.113566359	32	913	cellular lipid metabolic process
GO:0044710	3.92E-15	5.147019714	33.574394	72	5014	single-organism metabolic process
GO:0055114	1.26E-12	6.118728851	6.702825767	30	1001	oxidation-reduction process
GO:0006641	1.47E-12	20.18612254	0.816927816	13	122	triglyceride metabolic process
GO:0006639	3.35E-12	18.79565773	0.870496853	13	130	acylglycerol metabolic process
GO:0006638	3.70E-12	18.63510618	0.877192982	13	131	neutral lipid metabolic process
GO:0032787	3.07E-10	6.964980545	3.575733226	20	534	monocarboxylic acid metabolic process
GO:0046486	6.17E-10	8.751054852	2.22311504	16	332	glycerolipid metabolic process
GO:0019752	1.07E-09	5.210014948	6.140350877	25	917	carboxylic acid metabolic process
GO:0043436	2.65E-09	4.808991169	6.937190304	26	1036	oxoacid metabolic process
GO:0006066	3.07E-09	7.768745808	2.484264095	16	371	alcohol metabolic process
GO:0019915	3.32E-09	26.78623188	0.37498326	8	56	lipid storage
GO:0006082	3.73E-09	4.723571673	7.051024508	26	1053	organic acid metabolic process
GO:0055088	5.65E-09	15.29283489	0.783447168	10	117	lipid homeostasis
GO:0034308	7.59E-09	33.75920495	0.267845185	7	40	primary alcohol metabolic process
GO:0006631	3.57E-08	7.552344156	2.189634391	14	327	fatty acid metabolic process
GO:1901615	6.07E-08	5.811594894	3.495379671	17	522	organic hydroxy compound metabolic process
GO:0010883	1.28E-07	31.49787234	0.241060667	6	36	regulation of lipid storage
GO:0019432	1.58E-07	20.60135404	0.408463908	7	61	triglyceride biosynthetic process
GO:0046460	1.99E-07	19.86290323	0.421856167	7	63	neutral lipid biosynthetic process
GO:0046463	1.99E-07	19.86290323	0.421856167	7	63	acylglycerol biosynthetic process
GO:0010876	2.18E-07	7.047245858	2.149457613	13	321	lipid localization
GO:0008610	2.54E-07	5.220697074	3.863666801	17	577	lipid biosynthetic process
GO:0010889	6.02E-07	88.25595238	0.073657426	4	11	regulation of sequestering of triglyceride
GO:0042221	8.65E-07	2.744087825	24.57479577	47	3670	response to chemical
GO:0030730	1.80E-06	61.76666667	0.093745815	4	14	sequestering of triglyceride
GO:0051186	1.98E-06	6.244766275	2.203026651	12	329	cofactor metabolic process
GO:0006732	2.17E-06	6.847566967	1.834739521	11	274	coenzyme metabolic process
GO:0019433	2.49E-06	27.83082707	0.220972278	5	33	triglyceride catabolic process
GO:0044711	2.83E-06	3.190518521	10.49953127	27	1568	single-organism biosynthetic process
GO:1901700	2.93E-06	3.248058832	9.870095085	26	1474	response to oxygen-containing compound
GO:0006793	2.95E-06	2.726174825	19.13084237	39	2857	phosphorus metabolic process
GO:0045017	3.31E-06	7.346907994	1.546805946	10	231	glycerolipid biosynthetic process
GO:0010884	4.21E-06	47.50320513	0.113834204	4	17	positive regulation of lipid storage
GO:0046461	4.48E-06	24.34539474	0.247756797	5	37	neutral lipid catabolic process
GO:0046464	4.48E-06	24.34539474	0.247756797	5	37	acylglycerol catabolic process
GO:0048878	5.12E-06	3.670190275	6.468461229	20	966	chemical homeostasis
GO:0006091	6.78E-06	5.078345261	2.926208651	13	437	generation of precursor metabolites and energy
GO:0044283	8.01E-06	4.666883721	3.441810633	14	514	small molecule biosynthetic process
GO:0019637	8.08E-06	3.549692623	6.669345119	20	996	organophosphate metabolic process
GO:0019216	8.50E-06	6.561853351	1.720905317	10	257	regulation of lipid metabolic process
GO:0051235	1.10E-05	7.236593407	1.399491094	9	209	maintenance of location
GO:0016042	1.11E-05	6.352505447	1.774474354	10	265	lipid catabolic process
GO:0042572	1.26E-05	34.2962963	0.147314852	4	22	retinol metabolic process
GO:0046503	1.34E-05	18.98973042	0.308021963	5	46	glycerolipid catabolic process
GO:0006006	1.38E-05	7.022938227	1.439667872	9	215	glucose metabolic process
GO:0006094	1.45E-05	12.90673273	0.528994241	6	79	gluconeogenesis
GO:0006796	1.50E-05	2.54990826	18.8429088	37	2814	phosphate-containing compound metabolic process
GO:0006644	1.51E-05	5.517683665	2.249899558	11	336	phospholipid metabolic process
GO:0044242	1.75E-05	7.924925736	1.131645909	8	169	cellular lipid catabolic process
GO:0019319	1.80E-05	12.39473684	0.54908263	6	82	hexose biosynthetic process
GO:0044712	2.10E-05	3.211439287	7.734029731	21	1155	single-organism catabolic process
GO:0080184	2.38E-05	76.43298969	0.060265167	3	9	response to phenylpropanoid
GO:0046364	2.88E-05	11.34401436	0.595955538	6	89	monosaccharide biosynthetic process
GO:0010743	2.95E-05	26.83152174	0.1807955	4	27	regulation of macrophage derived foam cell differentiation
GO:0009725	2.98E-05	3.319650941	6.682737378	19	998	response to hormone
GO:0006650	3.18E-05	6.279789775	1.600374983	9	239	glycerophospholipid metabolic process
GO:0016051	4.06E-05	7.000477783	1.272264631	8	190	carbohydrate biosynthetic process
GO:0019318	4.38E-05	6.014010989	1.66733628	9	249	hexose metabolic process
GO:0006090	4.42E-05	10.45673759	0.642828445	6	96	pyruvate metabolic process
GO:0010742	4.54E-05	23.73076923	0.200883889	4	30	macrophage derived foam cell differentiation
GO:0055090	4.54E-05	23.73076923	0.200883889	4	30	acylglycerol homeostasis
GO:0070328	4.54E-05	23.73076923	0.200883889	4	30	triglyceride homeostasis
GO:0090077	4.54E-05	23.73076923	0.200883889	4	30	foam cell differentiation
GO:0051289	4.58E-05	14.40545809	0.395071649	5	59	protein homotetramerization
GO:0006069	4.62E-05	57.31701031	0.073657426	3	11	ethanol oxidation
GO:0015711	5.90E-05	4.713913018	2.611490558	11	390	organic anion transport
GO:0050872	6.13E-05	50.94501718	0.080353556	3	12	white fat cell differentiation
GO:0055089	6.13E-05	50.94501718	0.080353556	3	12	fatty acid homeostasis
GO:0010885	7.94E-05	45.84742268	0.087049685	3	13	regulation of cholesterol storage
GO:0006790	8.37E-05	4.944785276	2.249899558	10	336	sulfur compound metabolic process
GO:0044262	8.42E-05	5.500713027	1.814651132	9	271	cellular carbohydrate metabolic process
GO:1901698	8.91E-05	3.13398307	6.622472211	18	989	response to nitrogen compound
GO:0050873	9.46E-05	19.2734375	0.241060667	4	36	brown fat cell differentiation
GO:0006067	0.0001005	41.67666354	0.093745815	3	14	ethanol metabolic process
GO:0010878	0.0001005	41.67666354	0.093745815	3	14	cholesterol storage
GO:0010243	0.000102	3.20515733	6.08008571	17	908	response to organonitrogen compound
GO:0043434	0.0001039	3.844403024	3.803401634	13	568	response to peptide hormone
GO:0006637	0.0001044	11.95870445	0.468729075	5	70	acyl-CoA metabolic process
GO:0035383	0.0001044	11.95870445	0.468729075	5	70	thioester metabolic process
GO:0005996	0.000105	5.334798535	1.868220169	9	279	monosaccharide metabolic process
GO:0033197	0.000125	38.20103093	0.100441945	3	15	response to vitamin E
GO:0015980	0.0001287	4.680232558	2.370429892	10	354	energy derivation by oxidation of organic compounds
GO:0006112	0.000129	6.903091398	1.118253649	7	167	energy reserve metabolic process
GO:0006114	0.0001326	302.7142857	0.020088389	2	3	glycerol biosynthetic process
GO:0001523	0.000145	11.10075188	0.502209723	5	75	retinoid metabolic process
GO:0008203	0.000153	8.241881299	0.803535556	6	120	cholesterol metabolic process
GO:0006071	0.0001531	35.26011102	0.107138074	3	16	glycerol metabolic process

GO:0065008	0.0001704	2.20801097	21.83607875	38	3261	regulation of biological quality
GO:1902652	0.0001751	8.028914348	0.823623945	6	123	secondary alcohol metabolic process
GO:1901652	0.0001819	3.620259598	4.024373912	13	601	response to peptide
GO:0071285	0.000185	32.73932253	0.113834204	3	17	cellular response to lithium ion
GO:0072521	0.000191	3.822184665	3.5020758	12	523	purine-containing compound metabolic process
GO:0009636	0.0001914	6.454191033	1.191911075	7	178	response to toxic substance
GO:0016101	0.0001967	10.35719298	0.535690371	5	80	diterpenoid metabolic process
GO:0016125	0.0002367	7.572065889	0.870496853	6	130	sterol metabolic process
GO:0019400	0.0002611	28.64304124	0.127226463	3	19	alditol metabolic process
GO:0019401	0.000264	151.3469388	0.026784519	2	4	alditol biosynthetic process
GO:0009719	0.0002654	2.534377387	10.62675773	23	1587	response to endogenous stimulus
GO:0015909	0.0002707	14.33236434	0.314718093	4	47	long-chain fatty acid transport
GO:0033273	0.000276	9.586094867	0.575867149	5	86	response to vitamin
GO:0046474	0.0002763	6.05955335	1.265568501	7	189	glycerophospholipid biosynthetic process
GO:0019395	0.0003072	9.353836398	0.589259408	5	88	fatty acid oxidation
GO:0034440	0.0003411	9.13250774	0.602651667	5	90	lipid oxidation
GO:0002021	0.000355	25.45704467	0.140618722	3	21	response to dietary excess
GO:0006721	0.000359	9.025703794	0.609347797	5	91	terpenoid metabolic process
GO:0044699	0.0003677	3.49870801	81.82670416	94	12220	single-organism process
GO:0055081	0.0004004	12.83506944	0.348198741	4	52	anion homeostasis
GO:0010891	0.0004381	100.8911565	0.033480648	2	5	negative regulation of sequestering of triglyceride
GO:0033591	0.0004381	100.8911565	0.033480648	2	5	response to L-ascorbic acid
GO:0035634	0.0004381	100.8911565	0.033480648	2	5	response to stilbenoid
GO:0036155	0.0004381	100.8911565	0.033480648	2	5	acylglycerol acyl-chain remodeling
GO:1901564	0.0004916	2.305094085	13.31190572	26	1988	organonitrogen compound metabolic process
GO:0097006	0.0004968	12.07761438	0.36828713	4	55	regulation of plasma lipoprotein particle levels
GO:0015850	0.0005213	5.424916574	1.406187224	7	210	organic hydroxy compound transport
GO:0006805	0.0005279	6.46617755	1.011115575	6	151	xenobiotic metabolic process
GO:0042592	0.0005343	2.489743386	9.722780233	21	1452	homeostatic process
GO:0046942	0.0005527	4.690499195	1.861524039	8	278	carboxylic acid transport
GO:0033993	0.0005732	3.044164428	5.135931432	14	767	response to lipid
GO:0010033	0.0005845	2.13318121	18.16659971	32	2713	response to organic substance
GO:0071466	0.0005857	6.333812536	1.031203964	6	154	cellular response to xenobiotic stimulus
GO:0015849	0.0005931	4.637999681	1.881612428	8	281	organic acid transport
GO:0010226	0.000602	20.82286785	0.167403241	3	25	response to lithium ion
GO:0046320	0.000602	20.82286785	0.167403241	3	25	regulation of fatty acid oxidation
GO:0008654	0.0006335	5.241577061	1.453060131	7	217	phospholipid biosynthetic process
GO:0010890	0.0006543	75.66326531	0.040176778	2	6	positive regulation of sequestering of triglyceride
GO:0060159	0.0006543	75.66326531	0.040176778	2	6	regulation of dopamine receptor signaling pathway
GO:0014070	0.0006764	2.989738296	5.222981117	14	780	response to organic cyclic compound
GO:0006869	0.0007127	4.503481355	1.935181465	8	289	lipid transport
GO:0009410	0.0007161	6.084553744	1.071380742	6	160	response to xenobiotic stimulus
GO:0046128	0.0007795	4.439054157	1.961965984	8	293	purine ribonucleoside metabolic process
GO:0045834	0.0007868	7.527337762	0.723182001	5	108	positive regulation of lipid metabolic process
GO:0042278	0.0008327	4.391908213	1.982054373	8	296	purine nucleoside metabolic process
GO:0006654	0.0008451	18.32041237	0.18749163	3	28	phosphatidic acid biosynthetic process
GO:0046473	0.0008451	18.32041237	0.18749163	3	28	phosphatidic acid metabolic process
GO:0098754	0.0008451	18.32041237	0.18749163	3	28	detoxification
GO:0051262	0.0008549	7.382957393	0.73657426	5	110	protein tetramerization
GO:0006720	0.0008905	7.312810328	0.74327039	5	111	isoprenoid metabolic process
GO:0010886	0.0009121	60.52653061	0.046872907	2	7	positive regulation of cholesterol storage
GO:0042758	0.0009121	60.52653061	0.046872907	2	7	long-chain fatty acid catabolic process
GO:0044539	0.0009121	60.52653061	0.046872907	2	7	long-chain fatty acid import
GO:0046322	0.0009121	60.52653061	0.046872907	2	7	negative regulation of fatty acid oxidation
GO:0055098	0.0009121	60.52653061	0.046872907	2	7	response to low-density lipoprotein particle
GO:0006096	0.0009924	9.927419355	0.441944556	4	66	glycolytic process
GO:0034284	0.0010123	5.674661509	1.145038168	6	171	response to monosaccharide
GO:0006820	0.0010226	3.316208866	3.642694523	11	544	anion transport
GO:0006757	0.0010501	9.769179894	0.448640686	4	67	ATP generation from ADP
GO:0046890	0.0010855	6.981033665	0.776751038	5	116	regulation of lipid biosynthetic process
GO:0009119	0.0012171	4.128445581	2.102584706	8	314	ribonucleoside metabolic process
GO:0006898	0.001228	4.655822854	1.627159502	7	243	receptor-mediated endocytosis
GO:1901575	0.0012374	2.273812321	11.10887907	22	1659	organic substance catabolic process
GO:1903523	0.0012549	15.78919303	0.214276148	3	32	negative regulation of blood circulation
GO:0030301	0.0013043	9.183457711	0.475425204	4	71	cholesterol transport
GO:0005975	0.0013513	2.767836984	5.611356636	14	838	carbohydrate metabolic process
GO:0090407	0.0013673	3.191803645	3.776617115	11	564	organophosphate biosynthetic process
GO:0015918	0.001374	9.047794118	0.482121334	4	72	sterol transport
GO:0034637	0.001374	9.047794118	0.482121334	4	72	cellular carbohydrate biosynthetic process
GO:0006163	0.0013969	3.395744681	3.214142226	10	480	purine nucleotide metabolic process
GO:0048545	0.0014145	3.662890955	2.671755725	9	399	response to steroid hormone
GO:0015908	0.0014462	8.916062802	0.488817464	4	73	fatty acid transport
GO:0044282	0.0015143	3.982169798	2.176242132	8	325	small molecule catabolic process
GO:0006107	0.0015499	43.22740525	0.060265167	2	9	oxaloacetate metabolic process
GO:0006600	0.0015499	43.22740525	0.060265167	2	9	creatine metabolic process
GO:0050896	0.0015705	1.874247789	49.87277354	65	7448	response to stimulus
GO:0042493	0.0015944	3.596562128	2.718628633	9	406	response to drug
GO:0046031	0.0015986	8.663732394	0.502209723	4	75	ADP metabolic process
GO:0019217	0.0016789	8.542824074	0.508905852	4	76	regulation of fatty acid metabolic process
GO:0044723	0.0016831	2.942507264	4.479710727	12	669	single-organism carbohydrate metabolic process
GO:0009743	0.0017363	5.082099907	1.272264631	6	190	response to carbohydrate
GO:0055086	0.0017695	2.92387567	4.506495246	12	673	nucleobase-containing small molecule metabolic process
GO:0033500	0.0018308	5.02676733	1.28565689	6	192	carbohydrate homeostasis
GO:0042593	0.0018308	5.02676733	1.28565689	6	192	glucose homeostasis
GO:0044724	0.0018616	6.143692565	0.877192982	5	131	single-organism carbohydrate catabolic process
GO:0009116	0.0019016	3.833751817	2.256595688	8	337	nucleoside metabolic process
GO:0032371	0.0019189	13.46270467	0.247756797	3	37	regulation of sterol transport
GO:0032374	0.0019189	13.46270467	0.247756797	3	37	regulation of cholesterol transport
GO:0046173	0.0019189	13.46270467	0.247756797	3	37	polyol biosynthetic process
GO:0055091	0.0019289	37.82142857	0.066961296	2	10	phospholipid homeostasis
GO:0060100	0.0019289	37.82142857	0.066961296	2	10	positive regulation of phagocytosis, engulfment

GO:0009062	0.0020279	8.091008772	0.535690371	4	80	fatty acid catabolic process
GO:0006734	0.0020733	13.07717231	0.254452926	3	38	NADH metabolic process
GO:0006633	0.0021237	5.953036437	0.903977501	5	135	fatty acid biosynthetic process
GO:0032868	0.0021427	3.436276976	2.839158966	9	424	response to insulin
GO:0015718	0.0021933	5.907191643	0.910673631	5	136	monocarboxylic acid transport
GO:0006165	0.0022199	7.882478632	0.54908263	4	82	nucleoside diphosphate phosphorylation
GO:0009056	0.0022982	2.06466214	13.97482255	25	2087	catabolic process
GO:0006768	0.0023473	33.61678005	0.073657426	2	11	biotin metabolic process
GO:0034370	0.0023473	33.61678005	0.073657426	2	11	triglyceride-rich lipoprotein particle remodeling
GO:0034372	0.0023473	33.61678005	0.073657426	2	11	very-low-density lipoprotein particle remodeling
GO:0042953	0.0023473	33.61678005	0.073657426	2	11	lipoprotein transport
GO:0044872	0.0023473	33.61678005	0.073657426	2	11	lipoprotein localization
GO:0016052	0.0024118	5.773762765	0.93076202	5	139	carbohydrate catabolic process
GO:0008202	0.0025314	4.075428708	1.84813178	7	276	steroid metabolic process
GO:0060420	0.0025822	12.0423223	0.274541315	3	41	regulation of heart growth
GO:1901657	0.0027173	3.609069391	2.39051828	8	357	glycosyl compound metabolic process
GO:0046939	0.0027542	7.405120482	0.582563278	4	87	nucleotide phosphorylation
GO:0032096	0.0028045	30.25306122	0.080353556	2	12	negative regulation of response to food
GO:0032099	0.0028045	30.25306122	0.080353556	2	12	negative regulation of appetite
GO:0060099	0.0028045	30.25306122	0.080353556	2	12	regulation of phagocytosis, engulfment
GO:0009135	0.0032404	7.062739464	0.609347797	4	91	purine nucleoside diphosphate metabolic process
GO:0009179	0.0032404	7.062739464	0.609347797	4	91	purine ribonucleoside diphosphate metabolic process
GO:0006000	0.0033	27.50092764	0.087049685	2	13	fructose metabolic process
GO:0010745	0.0033	27.50092764	0.087049685	2	13	negative regulation of macrophage derived foam cell differentiation
GO:0016024	0.0033	27.50092764	0.087049685	2	13	CDP-diacylglycerol biosynthetic process
GO:0045821	0.0033	27.50092764	0.087049685	2	13	positive regulation of glycolytic process
GO:0046339	0.0033	27.50092764	0.087049685	2	13	diacylglycerol metabolic process
GO:0050746	0.0033	27.50092764	0.087049685	2	13	regulation of lipoprotein metabolic process
GO:0055094	0.0033	27.50092764	0.087049685	2	13	response to lipoprotein particle
GO:0048639	0.0033501	5.331760436	1.004419446	5	150	positive regulation of developmental growth
GO:0010038	0.0034593	3.842407093	1.955269854	7	292	response to metal ion
GO:0009185	0.0035042	6.903089888	0.622740056	4	93	ribonucleoside diphosphate metabolic process
GO:0097194	0.0035042	6.903089888	0.622740056	4	93	execution phase of apoptosis
GO:0009150	0.0036353	3.161318681	3.073523503	9	459	purine ribonucleotide metabolic process
GO:0034754	0.0036414	6.825925926	0.629436186	4	94	cellular hormone metabolic process
GO:0044763	0.0037224	2.117036155	74.4475693	86	11118	single-organism cellular process
GO:0010744	0.0038332	25.20748299	0.093745815	2	14	positive regulation of macrophage derived foam cell differentiation
GO:0034389	0.0038332	25.20748299	0.093745815	2	14	lipid particle organization
GO:0046341	0.0038332	25.20748299	0.093745815	2	14	CDP-diacylglycerol metabolic process
GO:0046459	0.0038332	25.20748299	0.093745815	2	14	short-chain fatty acid metabolic process
GO:0051194	0.0038332	25.20748299	0.093745815	2	14	positive regulation of cofactor metabolic process
GO:0051197	0.0038332	25.20748299	0.093745815	2	14	positive regulation of coenzyme metabolic process
GO:0070887	0.0040643	1.906685237	17.01486541	28	2541	cellular response to chemical stimulus
GO:0072329	0.0040748	6.604390681	0.649524575	4	97	monocarboxylic acid catabolic process
GO:0030813	0.0044037	23.26687598	0.100441945	2	15	positive regulation of nucleotide catabolic process
GO:0009259	0.0044229	3.062949981	3.167269318	9	473	ribonucleotide metabolic process
GO:0008217	0.0048928	4.857663025	1.09816526	5	164	regulation of blood pressure
GO:0010888	0.0050109	21.60349854	0.107138074	2	16	negative regulation of lipid storage
GO:0033194	0.0050109	21.60349854	0.107138074	2	16	response to hydroperoxide
GO:0033539	0.0050109	21.60349854	0.107138074	2	16	fatty acid beta-oxidation using acyl-CoA dehydrogenase
GO:0042136	0.0050109	21.60349854	0.107138074	2	16	neurotransmitter biosynthetic process
GO:0046500	0.0050109	21.60349854	0.107138074	2	16	S-adenosylmethionine metabolic process
GO:0019693	0.0051307	2.989728167	3.240926744	9	484	ribose phosphate metabolic process
GO:0019751	0.0053983	6.077970297	0.703093612	4	105	polyol metabolic process
GO:1901605	0.0054936	3.98254228	1.607071113	6	240	alpha-amino acid metabolic process
GO:0034383	0.0056544	20.16190476	0.113834204	2	17	low-density lipoprotein particle clearance
GO:0042180	0.0059449	3.914536027	1.633855631	6	244	cellular ketone metabolic process
GO:0007584	0.0061174	4.594611529	1.158430427	5	173	response to nutrient
GO:0030258	0.0061174	4.594611529	1.158430427	5	173	lipid modification
GO:0042445	0.0061174	4.594611529	1.158430427	5	173	hormone metabolic process
GO:0032095	0.0063336	18.9005102	0.120530333	2	18	regulation of response to food
GO:0035357	0.0063336	18.9005102	0.120530333	2	18	peroxisome proliferator activated receptor signaling pathway
GO:0009132	0.0063566	5.789308176	0.73657426	4	110	nucleoside diphosphate metabolic process
GO:0010676	0.0065615	8.465063001	0.381679389	3	57	positive regulation of cellular carbohydrate metabolic process
GO:0045444	0.0065696	4.513080948	1.178518816	5	176	fat cell differentiation
GO:0006127	0.0066961	Inf	0.00669613	1	1	glycerophosphate shuttle
GO:0009757	0.0066961	Inf	0.00669613	1	1	hexose mediated signaling
GO:0010182	0.0066961	Inf	0.00669613	1	1	sugar mediated signaling pathway
GO:0010255	0.0066961	Inf	0.00669613	1	1	glucose mediated signaling pathway
GO:0014843	0.0066961	Inf	0.00669613	1	1	growth factor dependent regulation of skeletal muscle satellite cell proliferation
GO:0018917	0.0066961	Inf	0.00669613	1	1	fluorene metabolic process
GO:0034633	0.0066961	Inf	0.00669613	1	1	retinol transport
GO:0038129	0.0066961	Inf	0.00669613	1	1	ERBB3 signaling pathway
GO:0038133	0.0066961	Inf	0.00669613	1	1	ERBB2-ERBB3 signaling pathway
GO:0042413	0.0066961	Inf	0.00669613	1	1	carnitine catabolic process
GO:0042443	0.0066961	Inf	0.00669613	1	1	phenylethylamine metabolic process
GO:0042941	0.0066961	Inf	0.00669613	1	1	D-alanine transport
GO:0046327	0.0066961	Inf	0.00669613	1	1	glycerol biosynthetic process from pyruvate
GO:0046359	0.0066961	Inf	0.00669613	1	1	butyrate catabolic process
GO:0070292	0.0066961	Inf	0.00669613	1	1	N-acylphosphatidylethanolamine metabolic process
GO:0071298	0.0066961	Inf	0.00669613	1	1	cellular response to L-ascorbic acid
GO:0072564	0.0066961	Inf	0.00669613	1	1	blood microparticle formation
GO:1901558	0.0066961	Inf	0.00669613	1	1	response to metformin
GO:1903280	0.0066961	Inf	0.00669613	1	1	negative regulation of calcium:sodium antiporter activity
GO:2000230	0.0066961	Inf	0.00669613	1	1	negative regulation of pancreatic stellate cell proliferation
GO:2000332	0.0066961	Inf	0.00669613	1	1	regulation of blood microparticle formation
GO:2000334	0.0066961	Inf	0.00669613	1	1	positive regulation of blood microparticle formation
GO:2000485	0.0066961	Inf	0.00669613	1	1	regulation of glutamine transport

GO:2000486	0.0066961	Inf	0.00669613	1	1	negative regulation of glutamine transport
GO:0033189	0.007048	17.78751501	0.127226463	2	19	response to vitamin A
GO:0010565	0.0072085	4.408721805	1.205303335	5	180	regulation of cellular ketone metabolic process
GO:0045471	0.0074219	5.526651652	0.770054908	4	115	response to ethanol
GO:0019674	0.0075639	8.017905589	0.401767778	3	60	NAD metabolic process
GO:0019362	0.0076482	5.476934524	0.776751038	4	116	pyridine nucleotide metabolic process
GO:0046496	0.0076482	5.476934524	0.776751038	4	116	nicotinamide nucleotide metabolic process
GO:0045922	0.0077971	16.79818594	0.133922593	2	20	negative regulation of fatty acid metabolic process
GO:0060419	0.0082797	7.745063778	0.415160037	3	62	heart growth
GO:0009117	0.0083616	2.599780702	4.138208116	10	618	nucleotide metabolic process
GO:0042744	0.0085805	15.91299678	0.140618722	2	21	hydrogen peroxide catabolic process
GO:0045979	0.0085805	15.91299678	0.140618722	2	21	positive regulation of nucleoside metabolic process
GO:1903580	0.0085805	15.91299678	0.140618722	2	21	positive regulation of ATP metabolic process
GO:0048638	0.0086166	3.606135576	1.767778224	6	264	regulation of developmental growth
GO:0006635	0.008652	7.615463918	0.421856167	3	63	fatty acid beta-oxidation
GO:0003012	0.0087083	3.208665402	2.323556984	7	347	muscle system process
GO:0046034	0.0088024	4.190503432	1.265568501	5	189	ATP metabolic process
GO:0031667	0.0088231	2.577669023	4.171688764	10	623	response to nutrient levels
GO:0071248	0.008848	5.241096866	0.810231686	4	121	cellular response to metal ion
GO:1990267	0.008848	5.241096866	0.810231686	4	121	response to transition metal nanoparticle
GO:0006753	0.0089177	2.573289902	4.178384894	10	624	nucleoside phosphate metabolic process
GO:0010035	0.0089955	2.919833789	2.926208651	8	437	response to inorganic substance
GO:0072524	0.0091018	5.196327684	0.816927816	4	122	pyridine-containing compound metabolic process
GO:0045981	0.0093604	5.152310924	0.823623945	4	123	positive regulation of nucleotide metabolic process
GO:1900544	0.0093604	5.152310924	0.823623945	4	123	positive regulation of purine nucleotide metabolic process
GO:0019430	0.0093976	15.11632653	0.147314852	2	22	removal of superoxide radicals
GO:0032098	0.0093976	15.11632653	0.147314852	2	22	regulation of appetite
GO:0045822	0.0093976	15.11632653	0.147314852	2	22	negative regulation of heart contraction
GO:1900117	0.0093976	15.11632653	0.147314852	2	22	regulation of execution phase of apoptosis
