# Supplementary material for: Transcriptome evolution from breast epithelial cells to basal-like tumors
Source: Oncotarget. 2017 Dec 8;9(1):453–63. doi: 10.18632/oncotarget.23065 (PMC5787480; doi:10.18632/oncotarget.23065)
Supplement: Supplementary file 4 [file oncotarget-09-453-s004.doc]

| **M1-GREEN** Biological Function: Angiogenesis | |
| --- | --- |
| **TOP 20 HUBS** | **DRUGS** |
| EFB1,AKAP12,GNG11, MRGPF, OLFML1,MMRN2,LHFP,ECSCR,  JAM2, ADGRA2,SLIT3,CNRIP1, LDB2,ZCCHC24,RUNX1T1,IGFBP6,  LRRN4CL,NDN, ANGPTL1,PEAR1 | No data |
| **TOP PPI DEGREE** | **DRUGS** |
| MEOX2,CAV1,TCF4 | No data |
| **M2-BROWN** Biological Function: Gland development | |
| **TOP 20 HUBS** | **DRUGS** |
| MLPH,ANKRD30A,FOXA1,AGR2, LINC00993,CAPN8,TFF1,C9ORF159,AGR3, LOC101926959, PRR15, SIDT1, TBC1D9,REEP6, ANXA9, DEGS2, DNAJC12 | No data |
| ERBB4 | DACOMITINIB/LAPATINIB/BMS-599626/ NERATINIB/ PELITINIB/AFATINIB/DIMALEATE/ DIMALEATE/GEFITINIB/ POZIOTINIB |
| CA12 | TOPIRAMATE/ HYDROFLUMETHIAZIDE/ HYDROCHLOROTHIAZIDE/ ELLAGIC ACID/ DICHLORPHENAMIDE/ BENZTHIAZIDE/ ACETAZOLAMIDE SODIUM/ ZONISAMIDE |
| AR | NILUTAMIDE/ BICALUTAMIDE/ FLUOXYMESTERONE/ FLUTAMIDE/ METHYLTESTOSTERONE/ OXANDROLONE/ TESTOSTERONE/ TESTOSTERONE PROPIONATE/ TESTOSTERONE PROPIONATE/ CALUSTERONE/ CYPROTERONE ACETATE/ DANAZOL/ ENZALUTAMIDE/ LEVONORGESTREL/ LEVONORGESTREL/ DROSPIRENONE/ DROSPIRENONE/ FLUDROCORTISONE/ METHYLTRIENOLONE/ NANDROLONE/ NANDROLONE DECANOATE/ (2S)-N-(4-CYANO-3-IODOPHENYL)-3-(4-CYANOPHENOXY)-2-HYDROXY-2-METHYLPROPANAMIDE/ (5S,8R,9S,10S,13R,14S,17S)-13-{2-[(3,5-DIFLUOROBENZYL)OXY]ETHYL}-17-HYDROXY-10-METHYLHEXADECAHYDRO-3H-CYCLOPENTA[A]PHENANTHREN-3-ONE/ 4-[(7R,7AS)-7-HYDROXY-1,3-DIOXOTETRAHYDRO-1H-PYRROLO[1,2-C]IMIDAZOL-2(3H)-YL]-1-NAPHTHONITRILE/ 4-{[(1R,2S)-1,2-DIHYDROXY-2-METHYL-3-(4-NITROPHENOXY)PROPYL]AMINO}-2-(TRIFLUOROMETHYL)BENZONITRILE/ ABIRATERONE/ CYPROTERONE/ DROMOSTANOLONE/ FLUFENAMIC ACID/ HYDROXYFLUTAMIDE/ KETOCONAZOLE/ SPIRONOLACTONE/ STANOZOLOL/ ANDARINE/ ARN-509/ ASC-J9/ BISPHENOL A/ ENOBOSARM/ EPALRESTAT/ GALETERONE |
| **TOP PPI DEGREE** | **DRUGS** |
| SPDEF,RAB27B | No data |
| **M3-DARKRED** Biological Function: Transport | |
| **TOP 20 HUBS** | **DRUGS** |
| CCDC144CP,MEFV,PGMSP2,OPHN1, FRG1, LOC101927769, LOC101928288, NEGR1, VAMP7, TMEM241, ANKRD20A5P, LOC101927305, ZNF595, ABHD3, DPY19L2P2, IL13RA1, | No data |
| LHCGR | GOSERELIN/ BUSERELIN/ CETRORELIX/ HCG/ AVICINE |
| IGF2R | ALPHA-D-MANNOSE-6-PHOSPHATE |
| MAP2K1 | TRAMETINIB/SELUMETINIB/AZD8330/BOSUTINIB/DB06892/ K-252A/TAK-733/ VEMURAFENIB/DABRAFENIB MESYLATE/GDC-0623/ MEK INHIBITOR I/MEK INHIBITOR II/PIMASERTIB/ REFAMETINIB/ RO4987655/SORAFENIB |
| **M4-TURQUOISE** Biological Function: Cell cycle | |
| **TOP 20 HUBS** | **DRUGS** |
| MAD2L1,TPX2, UBE2T, NUF2, ANLN,CDKN3,FAM83D,NCAPG,CDC20,MELK,KIF14,DLGAP5,CENPF,UBE2S,UBEC | No data |
| CCNA2 | (2S)-N-[(3Z)-5-CYCLOPROPYL-3H-PYRAZOL-3-YLIDENE]-2-[4-(2-OXOIMIDAZOLIDIN-1-YL)PHENYL]PROPANAMIDE/1-(3,5-DICHLOROPHENYL)-5-METHYL-1H-1,2,4-TRIAZOLE-3-CARBOXYLIC ACID/2-ANILINO-6 CYCLOHEXYLMETHOXYPURINE/3-(6-  CYCLOHEXYLMETHOXY-9H-PURIN-2-YLAMINO)-BENZENESULFONAMIDE/3-({2-[(4-{[6-(CYCLOHEXYLMETHOXY)-9H-PURIN-2-YL]AMINO}PHENYL)SULFONYL]ETHYL}AMINO)PROPAN-1-OL/4-(6-CYCLOHEXYLMETHOXY-9H-PURIN-2-YLAMINO)—BENZAMIDE/4-[(7-OXO-7H-THIAZOLO[5,4-E]INDOL-8-YLMETHYL)-AMINO]-N-PYRIDIN-2-YL-BENZENESULFONAMIDE/4-METHYL-5-{(2E)-2-[(4-MORPHOLIN-4-YLPHENYL)IMINO]-2,5-DIHYDROPYRIMIDIN-4-YL}-1,3-THIAZOL-2-AMINE/4-{[4-AMINO-6-(CYCLOHEXYLMETHOXY)-5-NITROSOPYRIMIDIN-2-YL]AMINO}BENZAMIDE/5-[5,6-BIS(METHYLOXY)-1H-BENZIMIDAZOL-1-YL]-3-{[1-(2-CHLOROPHENYL)ETHYL]OXY}-2-THIOPHENECARBOXAMIDE/6-CYCLOHEXYLMETHOXY-2-(3'-CHLOROANILINO) PURINE/HYDROXY(OXO)(3-{[(2Z)-4-[3-(1H-1,2,4-TRIAZOL-1-YLMETHYL)PHENYL]PYRIMIDIN-2(5H)-YLIDENE]AMINO}PHENYL)AMMONIUM/ N-[4-(2,4-DIMETHYL-THIAZOL-5-YL)-PYRIMIDIN-2-YL]-N',N'-DIMETHYL-BENZENE-1,4-DIAMINE |
| CDK1 | DINACICLIB/ALSTERPAULLONE/SU9516/HYMENIALDISINE/ INDIRUBIN-3'-MONOXIME/OLOMOUCINE/ALOISINE A/ AMINOPURVALANOL A/AT7519/AURORA KINASE-CDK INHIBITOR/BOHEMINE/CDK1 INHIBITOR/CDK1/2 INHIBITOR III/ CDK1/5 INHIBITOR/CDK4 INHIBITOR/ FLAVOPIRIDOL/RGB-286638/RONICICLIB |
| KIF2C | DB04395 |
| PBK | GEFITINIB/ OTS964 |
| TOP2A | AMONAFIDE/AMSACRINE/ BANOXANTRONE/ BECATECARIN/ CIPROFLOXACIN/ DAUNORUBICIN/ DAUNORUBICIN CITRATE/ DAUNORUBICIN HYDROCHLORIDE/ DEXRAZOXANE/ DOXORUBICIN/ DOXORUBICIN HYDROCHLORIDE/ ELSAMITRUCIN/ ENOXACIN/ EPIRUBICIN/ ETOPOSIDE/ ETOPOSIDE PHOSPHATE/ FINAFLOXACIN/ FLEROXACIN/ GENISTEIN/ IDARUBICIN/ IDARUBICIN HYDROCHLORIDE/ INNO-206/ LEVOFLOXACIN/ LOMEFLOXACIN/ LUCANTHONE/ MITOXANTRONE/ MITOXANTRONE HYDROCHLORIDE/ NORFLOXACIN/ OFLOXACIN/ PACLITAXEL/ PEFLOXACIN/ SPARFLOXACIN/ TENIPOSIDE/ TROVAFLOXACIN/ VALRUBICIN/ VORELOXIN |
| **TOP PPI DEGREE** | **DRUGS** |
| MCM2,PCNA | No data |
| AURKA | AT9283/ENMD-2076/ALISERTIB/PF-03814735/PHOSPHONOTHREONINE/ SNS-314/TOZASERTIB/4-(4-METHYLPIPERAZIN-1-YL)-N-[5-(2-THIENYLACETYL) -1,5-DIHYDROPYRROLO[3,4-C]  PYRAZOL-3-YL]BENZAMIDE/722544-51-6/ALISERTIB SODIUM/AMG 900/ AURORA KINASE INHIBITOR 3/AURORA KINASE INHIBITOR 2/CYC-116/ DANUSERTIB/MK-5108/MLN8054/RHO KINASE INHIBITOR 4/GSK-3 INHIBITOR 12/PACLITAXEL |
| CDK1 | DINACICLIB/HYMENIALDISINE/INDIRUBIN-3’-MONOXIME/OLOMUCINE/ ALOISINE A/AMINOPURVALANOLA/AT7519/AURORA KINASE/CDK INHIBITOR/BOHEMINE/CDK1 INHIBITOR/CDK1/2 INHIBITOR 3/CDK1/5 INHIBITOR/CDK4 INHIBITOR/FLAVOPIRIDOL/RGB-286638/RONICICLIB |
| **M5-YELLOW** Biological Function: Lipid metabolism | |
| **TOP 20 HUBS** | **DRUGS** |
| GPD1,CIDEC,TUSC5,LEPR, TIMP4, C14ORF180,PLIN4,MRAP,RBP4, ACVRVC,HSPB7,KLB,LIPE,LGALS12,  PLIN1,LOC1926901,GPAM,LVRN | No data |
| PCK1 | PHOSPHOENOLPYRUVATE/PHOSPHOMETHYLPHOSPHONIC ACID-GUANYLATE ESTER |
| SLC19A3 | L-CYSTEINE |
| **TOP PPI DEGREE** | **DRUGS** |
| ALDOC | No data |
| **M6-PURPLE** Biological Function: Lipid metabolism | |
| **TOP 20 HUBS** | **DRUGS** |
| CHMP2B,SMIM15,NRBF2TMEM251,  UBE2G1, RAD23B, YIPF5,PPP1R15B  NEK7,MITD1,DLD,TDP2,SPPL2A, DNAJB9,TEMEM14B,TMEM167A, KIF1BP,SRFBP1,MAP1LC3EB,EIF3J | No data |
| **M7-BLACK** Biological Function: Immune response | |
| **TOP 20 HUBS** | **DRUGS** |
| CD86,FYB,SAMSN1,TFEC,PTPRC, GBP5,PLEK,SLA,SLAMF8,GPR65, GZMB, MNDA,EPSTI1,CXCL11, LAPTM5,ADAMDEC1 | No data |
| CTSS | MORPHOLINE-4-CARBOXYLIC ACID [1-(2-BENZYLSULFANYL-1-FORMYL-ETHYLCARBAMOYL)-2-PHENYL-ETHYL]-AMIDE/(1R)-2-[(CYANOMETHYL)AMINO]-1-({[2-(DIFLUOROMETHOXY)BENZYL]SULFONYL}METHYL)-2-OXOETHYL MORPHOLINE-4-CARBOXYLATE/ DB08611/ N-(1-CYANOCYCLOPROPYL)-3-({[(2S)-5-OXOPYRROLIDIN-2-YL]METHYL}SULFONYL)-N~2~-[(1S)-2,2,2-TRIFLUORO-1-(4-FLUOROPHENYL)ETHYL]-L-ALANINAMIDE/ N-[(1S)-1-{1-[(1R,3E)-1-ACETYLPENT-3-EN-1-YL]-1H-1,2,3-TRIAZOL-4-YL}-1,2-DIMETHYLPROPYL]BENZAMIDE/ N-[(1S)-1-{1-[(1R,3E)-1-ACETYLPENT-3-EN-1-YL]-1H-1,2,3-TRIAZOL-4-YL}-1,2-DIMETHYLPROPYL]BENZAMIDE/ N-[(1S)-2-{[(1R)-2-(BENZYLOXY)-1-CYANO-1-METHYLETHYL]AMINO}-1-(CYCLOHEXYLMETHYL)-2-OXOETHYL]MORPHOLINE-4-CARBOXAMIDE/N-[1-(AMINOMETHYL)CYCLOPROPYL]-3-(MORPHOLIN-4-YLSULFONYL)-N~2~-[(1S)-2,2,2-TRIFLUORO-1-(4-FLUOROPHENYL)ETHYL]-L-ALANINAMIDE/ |
| PSMB9 | CARFILZOMIB/BORTEZOMIB |
| LCK | DB04395/ STAUROSPORINE/ {4-[2-ACETYLAMINO-2-(3-CARBAMOYL-2-CYCLOHEXYLMETHOXY-6,7,8,9-TETRAHYDRO-5H-BENZOCYCLOHEPTEN-5YLCARBAMOYL)-ETHYL]-2-PHOSPHONO-PHENYL}-PHOSPHONIC ACID/NINTEDANIB/PONATINIB/ ENMD-2076/LCK INHIBITOR/PAZOPANIB HYDROCHLORIDE/SARACATINIB/SU6656 |
| IL4I1 | VINYLGLYCINE |
| **TOP PPI DEGREE** | **DRUGS** |
| HLA-C,STAT1 | No data |
| ISG15 | IRINOTECAN |
| **M8-DARKTURQUOISE** Biological Function: Respiratory chain | |
| **TOP 20 HUBS** | **DRUGS** |
| STARD9,ACSM2A,MEG3, LMO7DN, NDUFAB1, VAMP8, SF3B4, UCHL3, NDUFA4, RPA3, LINC01111, TDRD10, GTF2IRD1, TRIM28, TDRD6, BCO2 | No data |
| SCN3B | ZONISAMIDE |
| ALDOA | 1,6-FRUCTOSE DIPHOSPHATE (LINEAR FORM) |
| MMP27 | MARIMASTAT |
| **M9-DARKGREY** Biological Function: Protein localization | |
| **TOP 20 HUBS** | **DRUGS** |
| KLHDC3,CALR,RNF19B,ACTB,CFL1, BANF1,ARHGDIA,GDI2, TMED2, HMGA1, UBE2M, SET, DRAP1, RAB5C | No data |
| YWHAE | FUSICOCCIN |
| TUBB | VINBLASTINE/COLCHICINE/VINCRISTINE/VINORELBINE/EPOTHILONE B/ALBENDAZOLE/CABAZITAXEL/CYT997/ DOCETAXEL/ERIBULIN/IXABEPILONE/MEBENDAZOLE/PACLITAXEL/ TASIDOTIN HYDROCHLORIDE |
| SLC25A39 | No data |
| PPP2R1A | (2S,3S,4E,6E,8S,9S)-3-AMINO-9-METHOXY-2,6,8-TRIMETHYL-10-PHENYLDECA-4,6-DIENOIC ACID/ 2,6,8-TRIMETHYL-3-AMINO-9-BENZYL-9-METHOXYNONANOIC ACID |
| HSP90AB1 | (5E)-12-CHLORO-13,15-DIHYDROXY-4,7,8,9-TETRAHYDRO-2-BENZOXACYCLOTRIDECINE-1,10(3H,11H)-DIONE/(5E)-14-CHLORO-15,17-DIHYDROXY-4,7,8,9,10,11-HEXAHYDRO-2-BENZOXACYCLOPENTADECINE-1,12(3H,13H)-DIONE/(5Z)-12-CHLORO-13,15-DIHYDROXY-4,7,8,9-TETRAHYDRO-2-BENZOXACYCLOTRIDECINE-1,10(3H,11H)-DIONE/ 2-(3-AMINO-2,5,6-TRIMETHOXYPHENYL)ETHYL 5-CHLORO-2,4-DIHYDROXYBENZOATE/CNF1010/ DB08464/ DB08464/SNX-5422/SNX-5422/AT13387/IPI-504/MPC-3100/RETASPIMYCIN/RETASPIMYCIN |
| FKBP1A | TEMSIROLIMUS/ EVEROLIMUS |
| **TOP PPI DEGREE** | **DRUGS** |
| ARRB2,PCBP1 | No data |
| YWHAE | FUSICOCCIN |
| HSP90AB1 | (5E)-12-CHLORO-13,15-DIHYDROXY-4,7,8,9-TETRAHYDRO-2-BENZOXACYCLOTRIDECINE-1,10(3H,11H)-DIONE / (5E)-14-CHLORO-15,17-DIHYDROXY-4,7,8,9,10,11-HEXAHYDRO-2-BENZOXACYCLOPENTADECINE-1,12(3H,13H)-DIONE / (5Z)-12-CHLORO-13,15-DIHYDROXY-4,7,8,9-TETRAHYDRO-2-BENZOXACYCLOTRIDECINE-1,10(3H,11H)-DIONE / 2-(3-AMINO-2,5,6-TRIMETHOXYPHENYL)ETHYL 5-CHLORO-2,4-DIHYDROXYBENZOATE / CNF1010/ DB08464/ GANETESPIB/ SNX-5422/ ALVESPIMYCIN/AT13387/ IPI-504/MPC-3100/RETASPIMYCIN/TANESPIMYCIN |
| **M10-CYAN** Biological Function: Protein ubiquitination | |
| **TOP 20 HUBS** | **DRUGS** |
| DMXL1,RNF111,ATP8B1,FAM179B,CETN3 | No data |
| **M15-ROYALBLUE** Biological Function: Metabolism and RNA processing | |
| **TOP 20 HUBS** | **DRUGS** |
| PFN1,RPS7,ERI3,TOMM20,SSB,MROL4,SNRPD2,ADRM1,RFXANK,SF3B5,BUD31,RAD23A,UFD1L,DNASE2,TALDO1,IMP4,GSTP1,ROMO1,COPS3,CWC15 | No data |
| GSTP1 | CLOMIPRAMINE/GLUTATHIONE SULFONIC ACID/ S-(P-NITROBENZYL)GLUTATHIONE/(9R,10R)-9-(S-GLUTATHIONYL)-10-HYDROXY-9,10-DIHYDROPHENANTHRENE/ 2-(N-MORPHOLINO)-ETHANESULFONIC ACID/CIBACRON BLUE/ GLUTATHIONE/ GLYCEROL/ CARBOPLATIN/CISPLATINUM/PACLITAXEL/ S-(4-BROMOBENZYL)CYSTEINE/S-NONYL-CYSTEINE/ 6-MERCAPTOPURINE/EZATIOSTAT/THIOTEPA |
| **TOP PPI DEGREE** | **DRUGS** |
| RPS7 | No data |
